# Herpesviruses shape tumour microenvironment through exosomal transfer of viral microRNAs

**DOI:** 10.1371/journal.ppat.1006524

**Published:** 2017-08-24

**Authors:** Ohad Yogev, Stephen Henderson, Matthew John Hayes, Sara Sofia Marelli, Yifat Ofir-Birin, Neta Regev-Rudzki, Javier Herrero, Tariq Enver

**Affiliations:** 1 UCL Cancer Institute, Research Department of Cancer Biology, Paul O’Gorman Building, University College London, London, England, United Kingdom; 2 UCL Cancer Institute, Bill Lyons Informatics Centre, Paul O’Gorman Building, University College London, London, England, United Kingdom; 3 UCL Institute of Ophthalmology, EM-Unit, Bath Street, London, England, United Kingdom; 4 Faculty of Biochemistry, Department of Biomolecular Sciences, Weizmann Institute of Science, Rehovot, Israel; University of Washington, UNITED STATES

## Abstract

Metabolic changes within the cell and its niche affect cell fate and are involved in many diseases and disorders including cancer and viral infections. Kaposi’s sarcoma-associated herpesvirus (KSHV) is the etiological agent of Kaposi’s sarcoma (KS). KSHV latently infected cells express only a subset of viral genes, mainly located within the latency-associated region, among them 12 microRNAs. Notably, these miRNAs are responsible for inducing the Warburg effect in infected cells. Here we identify a novel mechanism enabling KSHV to manipulate the metabolic nature of the tumour microenvironment. We demonstrate that KSHV infected cells specifically transfer the virus-encoded microRNAs to surrounding cells via exosomes. This flow of genetic information results in a metabolic shift toward aerobic glycolysis in the surrounding non-infected cells. Importantly, this exosome-mediated metabolic reprogramming of neighbouring cells supports the growth of infected cells, thereby contributing to viral fitness. Finally, our data show that this miRNA transfer-based regulation of cell metabolism is a general mechanism used by other herpesviruses, such as EBV, as well as for the transfer of non-viral onco-miRs. This exosome-based crosstalk provides viruses with a mechanism for non-infectious transfer of genetic material without production of new viral particles, which might expose them to the immune system. We suggest that viruses and cancer cells use this mechanism to shape a specific metabolic niche that will contribute to their fitness.

## Author summary

The metabolic state within a cell and its local environment is altered in many diseases and disorders including those caused by viral infections. The gamma-herpesviruses Kaposi’s Sarcoma Associated Herpesvirus (KSHV) is a viral agent associated with a large number of human malignancies. KSHV was shown to manipulate the metabolism of host cells and to induce similar metabolic changes to those found in non-viral cancers. Our work demonstrates that KSHV not only regulates host cell metabolism, but also alters the metabolism of neighbouring non-infected cells. We report that exosomes secreted from KSHV-infected cells selectively transfer the viral miRNAs into neighbouring cells. While these cells remain uninfected, the viral miRNAs are active in them to induce the Warburg effect. Moreover, our data show that this exosomal transfer of miRNAs transforms the exosome-recipient cells into ‘feeder cells’ producing a microenvironment that is more supportive for host cell growth. Finally, we found that this flow of genetic information increases the angiogenic potential of non-infected cells and therefore, could further enrich the infected cell microenvironment to support their fitness. This previously undescribed mechanism provides important insight into cancer and viral pathology and suggests new avenues for therapeutic intervention.

## Introduction

Altered metabolism is regarded as a hallmark of cancer. It is thought that cancer cells rewire metabolic pathways in such a way that biosynthetic processes are balanced against ATP production to support high rates of proliferation [1]. One of the most characteristic metabolic hallmarks of tumour metabolism is aerobic glycolysis. Despite the inefficiency of glycolysis in energy production, the glycolytic phenotype provides cancer cells with several advantages such as increased biosynthesis of intermediate macromolecules and anti-apoptosis and signalling through metabolites [2]. Recently, it has been suggested that cancer cells, in addition to their intrinsic metabolic alteration can also induce aerobic glycolysis in adjacent stromal cells, a phenomenon termed the ‘reverse Warburg Effect’ [3, 4]. The reverse Warburg effect emphasises the importance of tumour stromal cells in supplying energy metabolites and chemical building blocks to the rapidly proliferating cancer cells.

Oncogenic viruses cause more than 15% of human cancers and it is predicted that effective treatment against them will lead to 25% fewer cancers in developing countries and 7% in developed countries [5]. Pathogenicity of these viruses involves the hijacking of host cellular pathways, including those controlling cell metabolism, suggesting they can use as a model system to study cancer development.

Kaposi’s sarcoma herpesvirus (KSHV) is the etiological agent of Kaposi’s sarcoma (KS) and certain lymphoid neoplasms. KS is the most common neoplasm in HIV-1-infected individuals and also induces significant morbidity in other immunosuppressed individuals (e.g., post organ transplantation) and in populations where KSHV infection is endemic (para-Mediterranean regions) [6]. To date KSHV infection does not have an effective treatment and new therapeutic approaches are needed. KS cells, as other cancers, have a distinct metabolism and KSHV was shown to alter several metabolic pathways in its host cell [7–10]. We have recently shown that the KSHV-encoded microRNAs (miRNAs) induce aerobic glycolysis in infected cells through regulation of key cellular genes involved in mitochondrial activity and regulation of glucose metabolism [11]. Interestingly, interaction between KSHV infected cells and their microenvironment was shown to be important for primary effusion lymphoma growth *in vivo*[12]. It was recently shown that the KSHV miRNAs are present in exosomes isolated from KS patient and KS mouse model [13] but the role of this in KS biology is still unknown. We suggest that exosomes present a new possible platform allowing KSHV in infected cells to interact with their microenvironment and improve its host cell fitness.

Exosomes are spherical structures sealed with membranes released from the endosomal compartment of most cell types. They vary in size and molecular composition, depending on the cell of origin [14]. The functional impact of exosomes is imparted by the molecular components (protein and RNA cargo) they carry [15]. Exosome uptake is thought potentially to modulate many physiological and pathological processes including cell growth, immune regulation, angiogenesis and metastasis [16, 17]. Exosomes uptake was also shown to affect metabolism; exosomes secreted from cancer associated fibroblast were suggested to modulate cancer cells metabolism by transferring miRNAs and metabolites [18], and breast cancer secreted exosomes were shown to reduce glucose metabolism in cells in the pre-metastatic niche by transferring miR-122 [19]. Interestingly, it has been suggested that viruses, including KSHV, modulate the secretion of exosomes from infected cells [13, 20–22].

Here we have used KSHV infection as a model to study whether cells utilise the exosomal pathway to regulate the metabolism of their microenvironment. We show that KSHV-infected primary lymphatic endothelial cells secrete exosomes containing viral-encoded miRNAs. These exosomes transfer the miRNAs to surrounding uninfected cells to induce a reverse Warburg effect. We show that these miRNAs function in the recipient cells to regulate known target genes and this results in reduced mitochondria biogenesis and induction of aerobic glycolysis. Most importantly, we found that this metabolic cross-talk between infected and neighbouring non-infected cells has physiological implications in KSHV life cycle supporting the growth of latent infected cells. Finally, our results show that regulation of microenvironment metabolism by miRNA transfer might be a global mechanism used by other viruses and cancer cells.

Taken together our results reveal a novel mechanism whereby virally infected cells and cancer cells regulate the metabolism of surrounding cells via miRNA transfer. This allows these cells to control their microenvironment without producing new viral particles, which might trigger host immune recognition.

## Results

### Exosomes secreted from KSHV-infected cells contain viral miRNAs

KSHV miRNAs regulate mitochondria and glucose metabolism in infected cells [11]. In addition, it has been demonstrated that patient- and mouse model-derived exosomes carry in them the KSHV miRNAs [13]. We hypothesised, therefore, that KSHV modulates exosomes secretion to regulate the metabolism of neighbouring cells.

**Fig 1:**
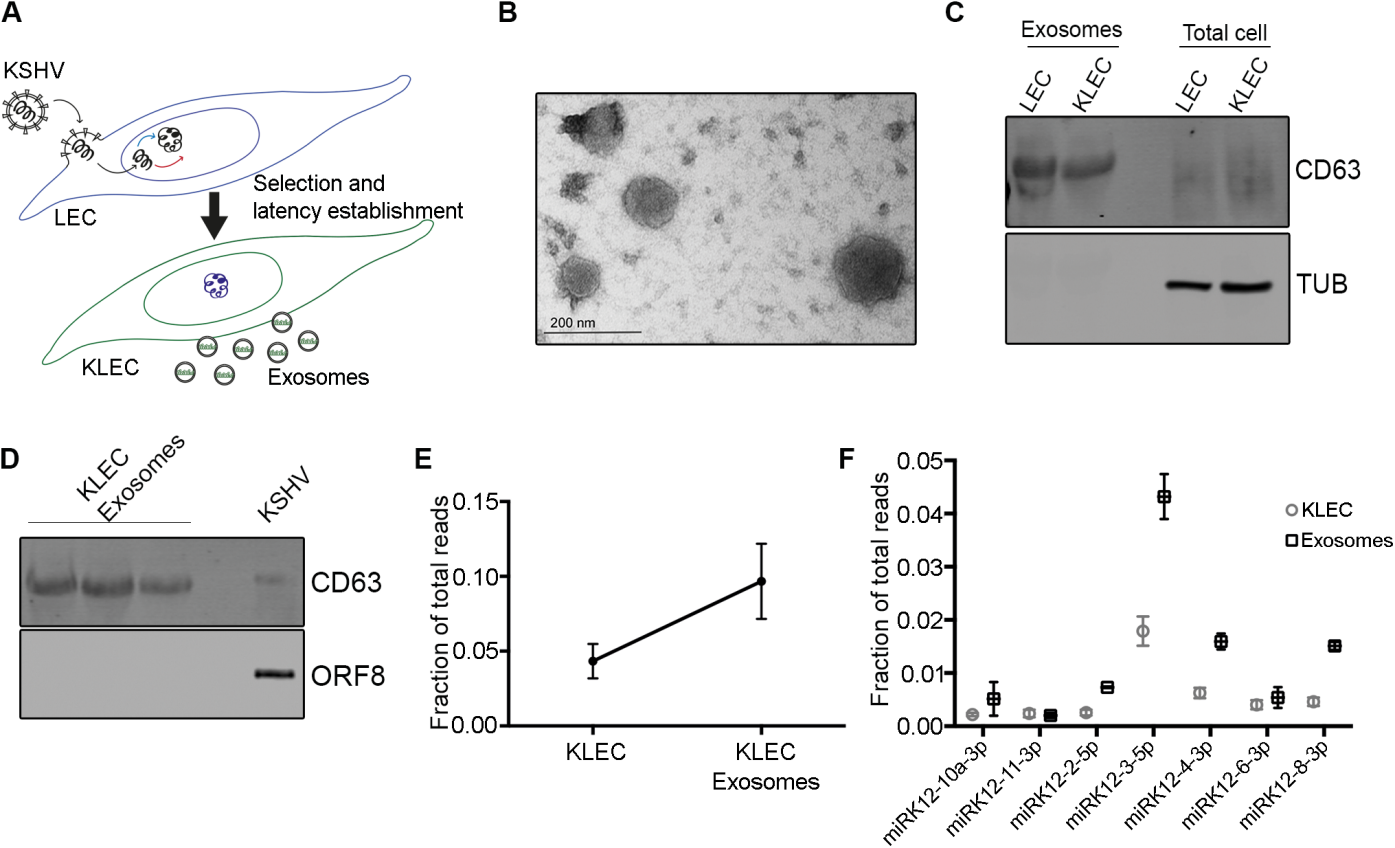
Characterisation of exosomes secreted from KLEC. **(A)** Schematic illustration of the procedure used in this study to collect exosomes from KLEC. **(B)** Exosomes were purified from KLEC and LEC growth media and analysed by electron microscopy (TEM). Representative image of exosomes from KLEC is shown. **(C)** Lysates from purified exosomes or whole cells were separated by SDS/PAGE and analysed by immunoblot for expression of the exosomal marker CD63. **(D)** Lysates from purified exosomes or KSHV (10μg) were separated by SDS/PAGE and analysed by immunoblot for expression of the exosomal marker CD63 (top panel) and the viral protein ORF8 (bottom panel). **(E)** Percentage of the viral miRNAs reads from the total reads detected. **(F)** Expression levels of selective KSHV miRNAs in KLEC and KLEC-derived exosomes. The expression level was calculated as fraction of total reads detected in KLEC and KLEC-derived exosomes. Both graphs present the mean and standard error of 3 biological repeats.

We test our hypothesis in lymphatic endothelial cells (LEC), the precursor cells for KS. We infected primary LEC with BAC16-derived WT KSHV (KLEC) or miRNA cluster-deleted KSHV (ΔmiR-KLEC) [23]. Cells were selected to produce a 100% infected population and cultured for 6 days to allow establishment of latency (Fig 1A and S1A Fig). Post selection, both KLEC and ΔmiR-KLEC were found to have similar KSHV genome copy number (S1B Fig) and expression levels of other latent genes (S1C Fig). Growth media was then collected every 48 hours for a period of 14 days after each infection and extracellular vesicles were purified by standard differential centrifugation from 150ml of media (S1D Fig). To determine if these nano-vesicles are exosomes we performed imaging, particle sizing, and measured biochemical properties of purified vesicles released from infected and non-infected LEC. Size and population characterisation using NanoSight Nanoparticle Tracking Analysis technology measured particles with diameter distribution between 50-150nm, corresponding to the expected size of exosomes (S1E Fig). This analysis also showed that 150ml of growth media contains between 2-3x10^11^ particles/ml (S1F Fig). Purity of the exosome preparations was determined by both electron microscopy (Fig 1B) and immunoblot analysis for the known exosome markers CD63, CD9 and ALIX (Fig 1C and S1G Fig).

Because our hypothesis is a non-cell autonomous regulation on cell metabolism, we confirmed that our purified exosomes are not contaminated with KSHV particles. Immunoblot analysis showed no detectable KSHV envelope-associated protein ORF8 in the purified exosome fraction (Fig 1D, bottom panel, 3 left lanes) and no viral particles were identified by electron microscopy. As expected, CD63 can be detected also in the KSHV sample (Fig 1D, top panel, last lane) since it is collected from the media of activated cells using high speed centrifugation. Finally, after incubation with purified exosomes, we could not detect the KSHV DNA in uninfected cells, and these cells did not become GFP positive nor kanamycin resistant, as would be expected had they become infected with the recombinant virus. Taken together, we concluded that the purified exosome fraction is KSHV free and therefore any effect this fraction has, is not due to *de novo* KSHV infection.

To test whether the KSHV miRNAs are present in secreted exosomes, RNA was extracted from LEC-, KLEC- and ΔmiR-KLEC-derived exosomes. To ascertain that the RNA is protected within the vesicles, exosomes were treated with 0.4μg/μl RNase for 10 min at 37°C prior to RNA extraction [24]. qRT-PCR analysis using the KSHV-miR LNA PCR primer sets (Exiqon), showed all 12 KSHV encoded miRNAs are present in KLEC derived exosomes (S1H Fig). Importantly, using these primer sets we could not detect any signal for RNA extracted from exosomes derived from LEC. Similarly, we could only detect miR-K12-10 and miR-K12-12, which are encoded out of the miR-cluster, in exosomes derived from ΔmiR-KLEC (S1I Fig). To better quantify the viral miRNAs in these exosomes as well as the effect of KSHV infection on transfer of cellular miRNAs in exosomes, we sequenced the small RNAs from LEC and KLEC as well as from exosomes secreted from these cells. We detected around 1800 different miRNAs in LEC and KLEC and around 1200 miRNAs in exosomes secreted from these cells. Importantly, we found that while in KLEC, the viral miRNAs present around 5% of the total miRNAs reads, in exosomes secreted from them, these miRNAs are responsible for around 10% of the total miRNAs reads (Fig 1E). In addition, we found differences in the expression profile of the viral miRNAs between the cells and exosomes. For example, KSHV miR-K12-10a-3p, K12-4-3p and K12-8-3p are over represented in the exosomes while KSHV miR-K12-11-3p and K12-4-5p are underrepresented (Fig 1F and S1 Table). Moreover, specific cellular miRNAs that are over represented in KLEC compared to LEC such as miR-145-5p and 143-3p (S2 Table), were not found to be enriched in KLEC-derived exosomes (S3 Table). On the other hand, we identified cellular miRNAs which are enriched in KLEC-derived exosomes compared to LEC-derived exosomes although these are not enriched in the respective cells (S2 and S3 Tables). For example, hsa-miR-216a is highly enriched in KLEC derived exosomes. miR-216a was suggested to function as an oncomiR and to induce epithelial-mesenchymal transition (EMT) by targeting PTEN and SMAD7 [25]. This suggests that transfer of human miRNAs in exosomes secreted from KSHV infected cells may play an additional role in KSHV pathogenicity. Taken together these results suggest that KSHV manipulates its host cells secretion system to selectively enrich the packaging of the viral miRNAs together with specific cellular miRNAs in exosomes secreted from infected cells.

### KSHV miRNAs are transferred to non-infected cells, where they are active

**Fig 2:**
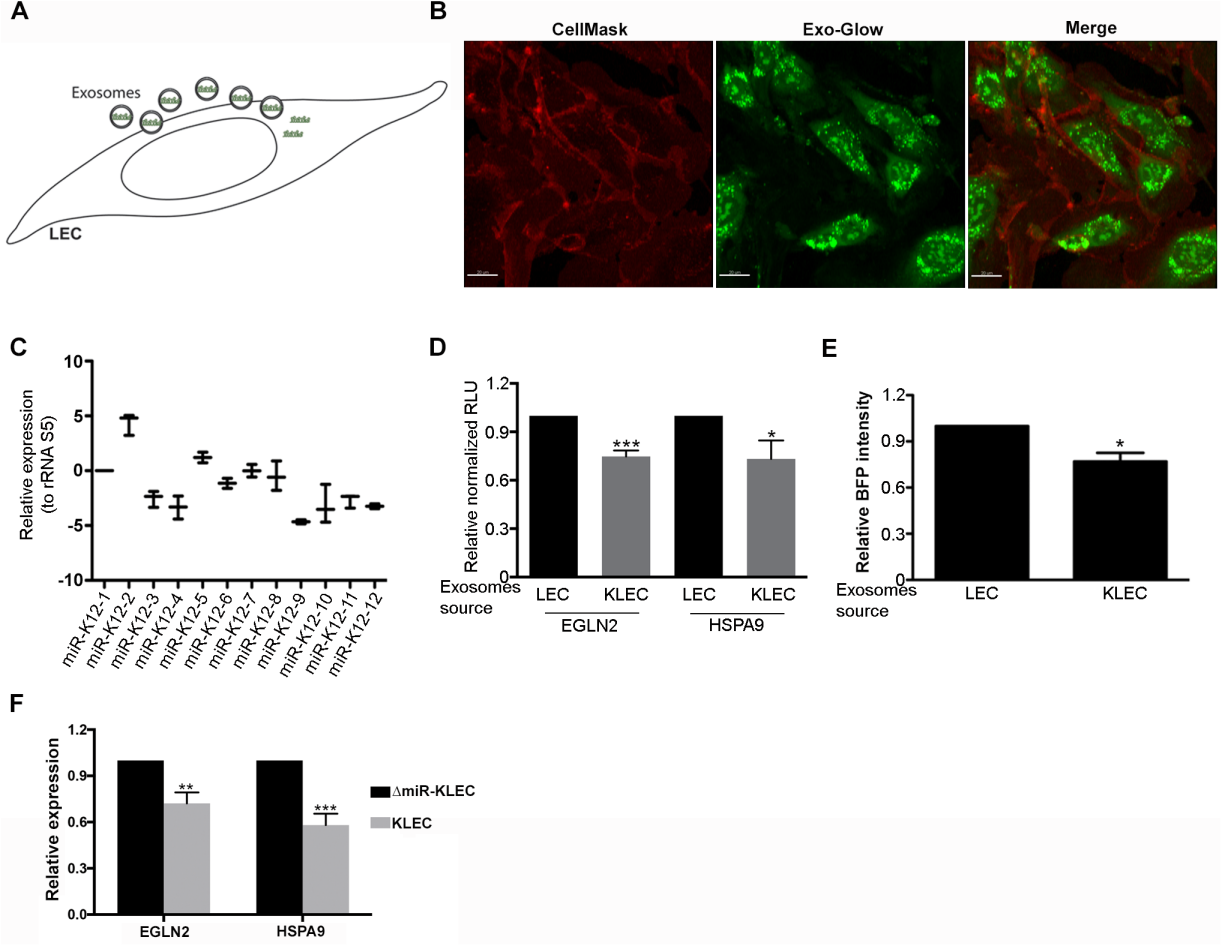
KSHV miRNAs are transferred via exosomes to naive cells to regulate gene expression. **(A)** Schematic illustration of exosomes education—10^5^ non-infected cells were incubated for total of 48 hours when 2.5x10^9^ exosomes were added every 24 hours. **(B)** KLEC exosomes are being taken up by naive LEC. LEC were first stained using CellMask deep red, then incubated with GFP labelled exosomes and analysed using a confocal microscope. **(C)** LEC were educated using 2.5x10^9^ KLEC-derived exosomes and tested for the presence of the mature KSHV miRNAs using the KSHV-miR LNA PCR primer sets (Exiqon) relative to the ribosomal RNA S5. **(D)** Reporter assay indicating the sensitivity of the EGLN2 or HSPA9 3’UTRs to targeting by the KSHV miRNAs. Cells expressing the reporter vectors were educated using LEC or KLEC-derived exosomes and analysed for luciferase activity. Firefly expression of each sample was internally normalized to Renilla expression to give the relative light units (RLU), which are presented relative to cells educated using LEC exosomes. **(E)** Reporter assay indicating miR-K12-10 is active in recipient cells. Cells expressing a blue fluorescent protein (BFP) fused to 8 repeats of the miR-K12-10 targeting sequence were educated using LEC or KLEC-derived exosomes and analysed for BFP expression by fluorescence-activated cell sorter (FACS). **(F)** Relative mRNA levels of EGLN2 and HSPA9 in LEC educated using KLEC-derived exosomes compared to LEC educated using ΔmiR-KLEC-derived exosomes. mRNA levels were determined by qRT-PCR. TUBB levels were used for normalisation. All panels present the mean and standard deviation of 3 biological repeats. Statistical significance was denoted by *P < .05, **P < .01, ***P < .001.

Having established the presence of KSHV miRNAs in exosomes we sought to explore their trafficking and more importantly, biological function. We initially tested whether exosomes secreted from KLEC are taken up by non-infected LEC, by labelling internal protein in purified exosomes using the Exo-Green (System Bioscience). Untreated LEC were first labelled using CellMask Deep Red Plasma Membrane Stain (molecular probes), then incubated with labelled exosomes and analysed using confocal microscopy. As shown in Fig 2B, upon incubation with labelled exosomes, GFP signal can be detected within the cells, indicating uptake of these exosomes. Flow cytometry analysis of these cells showed that positive staining is still detectable 24 hours’ post uptake by target cells (S2A Fig). Importantly, uptake of KLEC-derived exosomes results in transfer of the viral miRNAs to these cells (Fig 2C).

miRNAs function by binding to their target genes to inhibit their translation and induce their mRNA degradation. Therefore, we next tested if the KSHV miRNAs are active in the cytosol of recipient cells to inhibit the expression of known target genes. We have previously shown that the KSHV miRNAs function as cluster to regulate EGLN2 and HSPA9 and induce aerobic glycolysis [11]. We inserted the 3’ UTRs of these genes downstream of a luciferase coding sequence to generate a reporter of KSHV miRNA activity. Cells expressing this reporter were incubated with exosomes, secreted from LEC or KLEC for 48 hours (1x10^9^ particles). Incubation with KLEC-derived exosomes resulted in a ~25–30% reduction in luciferase activity relative to cells incubated with control LEC-secreted exosomes (Fig 2D). To further confirm the activity of the KSHV miRNAs in receptive cells we specifically tested the activity of miR-K12-10, which is expressed separately from the other miRNAs, using blue fluorescent protein (BFP) fused to 8 repeats of the miRNA target site. When these cells were incubated with KLEC-secreted exosomes, we found a ~25% reduction in BFP intensity compared to incubation with exosomes secreted from LEC (Fig 2E). To further test if these KLEC-derived exosomes regulate the expression of these genes under more physiological exosomes-transfer conditions we used Transwell plates [26] to co-culture non-infected LEC (bottom compartment) with either ΔmiR-KLEC or KLEC (upper compartment). We then analysed the RNA from the non-infected LEC and found 30% and 40% down regulation of EGLN2 and HSPA9 mRNAs levels respectively (Fig 2F). Taken together these results show that the KSHV encoded miRNAs are transferred to non-infected cells and maintain their ability to down-regulate specific target genes.

### Transfer of KSHV miRNAs via exosomes induces a reverse Warburg effect

**Fig 3:**
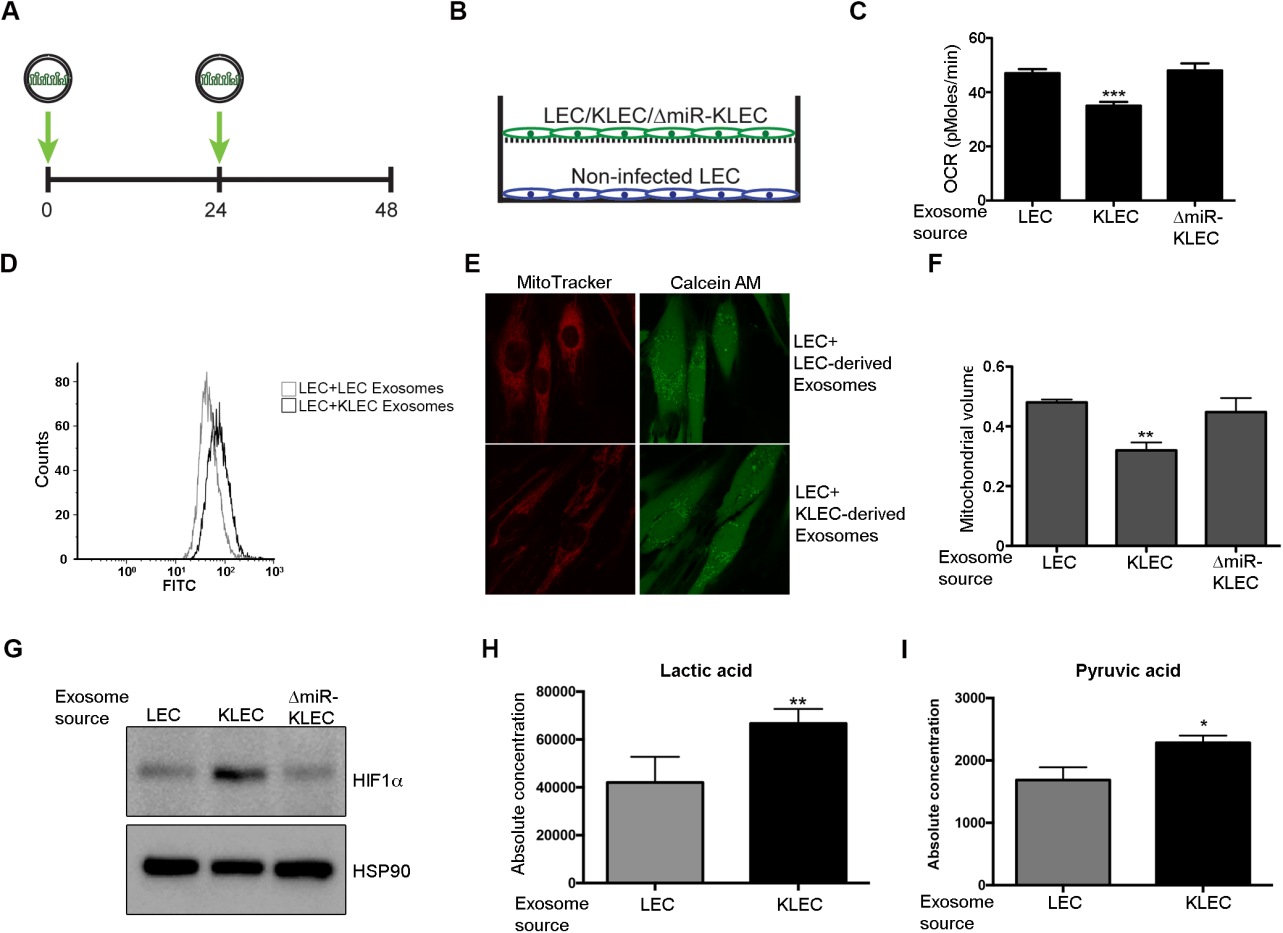
KLEC-derived exosomes induce the reverse Warburg effect. **(A-B)** Schematic illustration of exosome education. 10^5^ non-infected cells were incubated for total of 48 hours when 2.5x10^9^ exosomes were added every 24 hours (A) or co-cultured for 5 days with 10^5^ of the indicated cell in Transwell plates (B). **(C-G)** 10^5^ cells were educated using 2.5x10^9^ of the indicated exosomes and analysed for the following metrics: **(C)** Oxygen consumption rate (OCR) as measured using the Seahorse XF24 Analyser. Cells were seeded at a density of 4x104 cells per well and the assay was performed according to the manufacturer’s Mito stress protocol. The bar graph presents the average base line OCR from 3 independent experiments relative to non-targeting control OCR (mean+SD, n = 3). **(D)** Glucose uptake as measured by uptake of the fluorescent glucose analogue 6-NBDG. Cells were incubated with 30 μM of 6-NBDG for 20 minutes prior to analysis by fluorescence-activated cell sorter (FACS). The histogram displays one representative experiment. **(E-F)** Mitochondria volume—Cells were loaded with 5μM Calcein-AM and 5nM MitoTracker Deep Red FM and optical z-series were captured. Maximal projections of images were used to quantify the area of green (Calcein) and red (MitoTracker Deep Red) signals as previously described [11]. Representative single-plane images of the mitochondrial structure are shown on the left panel (E). The bar graph presents the average mitochondrial volume in cells from 3 different experiments (at least 10 fields per experiment) (F). **(G)** HIF1α protein expression, as measured by immunoblot. **(H-I)** Lactic acid (H) and pyruvic acid (I) concentrations as measured in educated cells using CE-TOFMS and CE-QqQMS (Human Metabolome Technologies, Inc.). All panels present the mean and standard deviation of 3 biological repeats. Statistical significance was denoted by *P < .05, **P < .01, ***P < .001.

We have previously shown that the KSHV miRNAs induce aerobic glycolysis in infected cells. We therefore predicted that they would have a similar effect in exosome recipient cells. To test this hypothesis, we educated non-infected LEC by either growing them in the presence of isolated exosomes for 48 hours (Fig 3A) or by co-culturing them with infected cells using Transwell plates (Fig 3B). We first measured the oxygen consumption rate of educated LEC using the Seahorse XF24 Analyzer. The Seahorse Extracellular Flux Analyzer determines oxygen consumption rate (OCR) and extracellular acidification rate (ECAR), in order to assess cellular functions such as oxidative phosphorylation and glycolysis. We educated non-infected cells with increasing numbers of exosomes and found a dose dependent reduction in oxygen consumption of educated cells with maximal effect using 2.5x10^9^ exosomes (S3A Fig). We therefore decided to use this number of exosomes for cell education for all future experiments.

Education using KLEC-derived exosomes reduced the baseline oxygen consumption in recipient cells by ~30% compared to education using LEC-derived exosomes (Fig 3C and S3A Fig). Importantly, exosomes derived from ΔmiR-KLEC did not affect oxygen consumption (Fig 3C), suggesting the viral miRNAs are the driving force behind this phenotype. Similarly, after co-culturing KLEC and LEC for 5 days, LEC were found to have reduced oxygen consumption compared to uneducated LEC (S3B Fig). This suggests that transfer of the KSHV miRNAs into non-infected cells reduces mitochondrial respiration. Similarly, we also observed a 30% increase in glucose uptake, consistent with increased aerobic glycolysis (Fig 3D). Mitochondria are key players in normal glucose metabolism during aerobic conditions and as part of the Warburg effect, many cancer types show altered mitochondrial activity [27]. Since we have previously shown that expression of the KSHV-encoded miRNAs reduces mitochondria biogenesis [11], we tested whether KLEC-derived exosomes have a similar effect on mitochondrial volume by loading cells with MitoTracker together with Calcein AM. MitoTracker is a fluorescent dye that labels mitochondria within live cells utilising the mitochondrial membrane potential. It therefore allowed us to calculate mitochondrial volume (MitoTracker staining) relative to total cell volume (Calcein staining) (Fig 3E). Upon incubation with KLEC-derived exosomes we found a ~40% decrease in mitochondria volume in educated cells compared to cells incubated with LEC derived exosomes (Fig 3F). Importantly, we did not detect any significant difference between cells educated using LEC- and ΔmiR-KLEC-derived exosomes, strengthening our notion that the KSHV encoded miRNAs are responsible for this phenotype. The hypoxia-induced factor alpha (HIF1α) is a known regulator of glucose metabolism [28, 29] and can mediate the Warburg effect in cancer cells [30]. KSHV has been shown to activate HIF1α and HIF2α during latency [31] and we have observed that expression of KSHV miRNAs induces HIF1α stabilisation [11]. As shown in Fig 3G, HIF1α expression was increased in cells educated using KLEC-derived exosomes compared to those educated using LEC- or ΔmiR-KLEC-derived exosomes. To further characterise the metabolic effect induced by exosomes secreted from KSHV infected cells, we performed targeted quantitative analysis using capillary electrophoresis mass spectrometry (CE-MS). This analysis showed significant increase in lactate and pyruvate as well as decrease levels of TCA cycle metabolites and ATP, in cells educated by KLEC derived exosomes (Fig 3H and 3I and S3C Fig), supporting our notion that these exosomes reduce mitochondrial activity in educated cells.

Importantly, when educated cells were grown for additional 5 days without exosomes, the metabolic phenotype was reversed and their oxygen consumption was comparable to that of untreated LEC (S3D Fig). This supports our notion that these cells are not infected by KSHV and that this phenotype depends on constant transfer of miRNAs from infected cells.

Finally, we tested whether exosomes secreted from KLEC have the same metabolic effect on other cell types relevant to KS. Education of Human Umbilical Vein Endothelial Cells (HUVEC), with exosomes extracted from KLEC induced aerobic glycolysis, as shown by reduced oxygen consumption and mitochondria volume (S3E and S3F Fig).

Taken together these results suggest that transfer of the KSHV miRNAs via exosomes induces aerobic glycolysis, reduces mitochondria biogenesis and leads to HIF1α stabilisation in surrounding non-infected cells.

### Exosomes secreted from EBV infected cells reduce mitochondrial respiration

**Fig 4:**
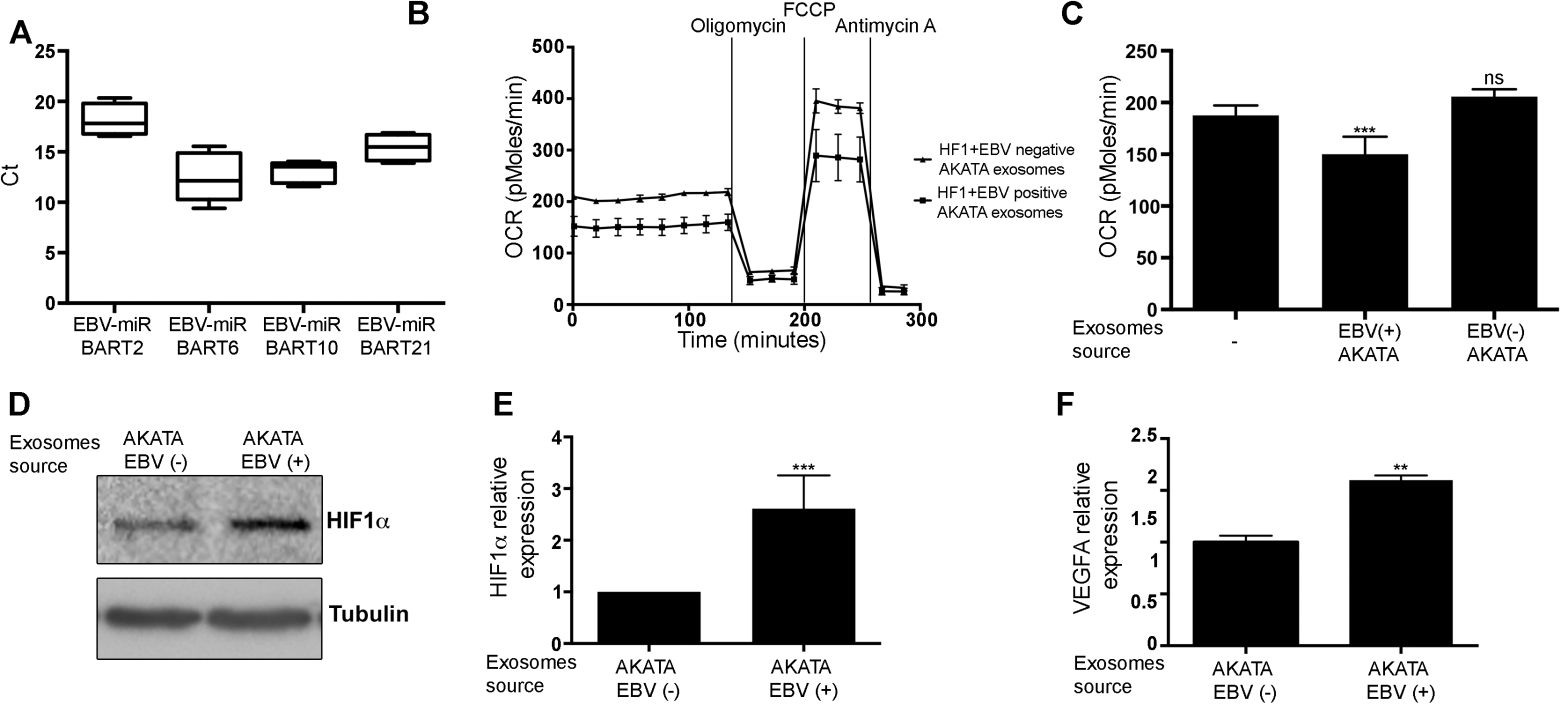
Exosomes secreted from EBV infected cells transfer the virus miRNAs and induce reverse Warburg effect. **(A)** Expression of 4 mature EBV miRNAs in exosomes purified from AKATA growth media. Detection of the mature EBV miRNAs was performed using the EBV-miR LNA PCR primer sets (Exiqon). **(B-C)** Oxygen consumption rate (OCR) as measured using the Seahorse XF24 Analyser. 10^5^ human fibroblasts cells were educated using 2.5x10^9^ of the indicated exosomes. Cells were seeded at a density of 4x10^4^ cells per well and the assay was performed according to the manufacturer’s Mito stress protocol. Representative Mito stress assay of the mitochondrial structure is shown on the left panel (B). The bar graph presents the average basal oxygen consumption of 3 biological repeats (C). **(D-E)** HIF1α protein expression, in human fibroblasts cells educated using the indicated exosomes, as measured by immunoblot (D). The bar graph presents the average relative expression in 3 independent experiments (E). **(F)** Expression of the HIF1α target gene VEGFA. mRNA levels were determined by quantitative real-time PCR (qRT-PCR). Tubulin beta (TUBB) levels were used for normalisation. In all panels, except to panel B, the graphs present the mean and standard deviation of 3 biological repeats. Statistical significance was denoted by *P < .05, **P < .01, ***P < .001.

Many viruses other than KSHV express miRNAs, and we speculated that miRNA transfer via exosomes might be a general mechanism used by viruses to regulate their microenvironment. Epstein Barr Virus (EBV) is a human gamma herpes virus which, like KSHV, is the etiological agent for several lymphoid malignancies [32]. EBV encodes at least 40 miRNAs, which were shown to be present in exosomes secreted from EBV transform cells [21, 33]. The EBV encoded miRNAs are thought to have many target genes in common with KSHV [34–36] and EBV-miR-BART1 has been suggested to regulate metabolism-associated genes [37]. To test whether exosomes secreted from EBV infected cells have similar effect to those secreted from KSHV infected cells, we collected exosomes from the growth media of EBV positive and negative AKATA cell lines (S4A and S4B Fig). We have found that EBV-encoded miRNAs were present in only exosomes secreted by the EBV positive AKATA cells (Fig 4A). Educating human fibroblasts using these exosomes for 48 hours resulted in a 25% decrease in oxygen consumption (Fig 4B), stabilisation of HIF1α (Fig 4C and 4D) and expression of its target gene VEGFA (Fig 4E). This suggests EBV can also use exosomes to alter its microenvironment metabolism in a similar way to KSHV.

### The hypoxia-induced miR-210 can be transferred in exosomes to regulate microenvironment metabolism

Regulating energy metabolism using miRNAs is not exclusive to viruses, and many cellular miRNAs are also known to control energy metabolism [38]. miR-210, for example, is known to regulate cell metabolism, is associated with mitochondrial defects and glycolytic phenotype [39–41], and was suggested to be secreted in exosomes under hypoxic conditions [42]. To test if miR-210 can be transferred in exosomes to alter the metabolism of cells within the microenvironment we forced the expression of miR-210 in HEK293T and HCT116 cell lines. Exosomes secreted from these cells had much higher levels of miR-210 compared to exosomes derived from cells infected with a control vector (S5A Fig). miR-210 directly targets the iron-sulphur assembly proteins ISCU1/2 [43]. To test if miR-210 can be transferred in exosomes and be active in recipient cells, we tested ISCU1 mRNA levels in human fibroblasts, educated using exosomes secreted from control or miR-210 over expressing cells. Education using exosomes contain high levels of miR-210 leads to a ~50% decrease of ISCU1 mRNA levels (S5B Fig). Importantly, these educated cells also reduced their oxygen consumption by 30–40% (S5C Fig).

Taken together these results suggest that transfer of miRNAs via exosomes is a general mechanism that can be used by cells in a variety of pathological contexts to regulate the metabolism of cells in their microenvironment.

### Glycolytic cells are less sensitive to KSHV infection

**Fig 5:**
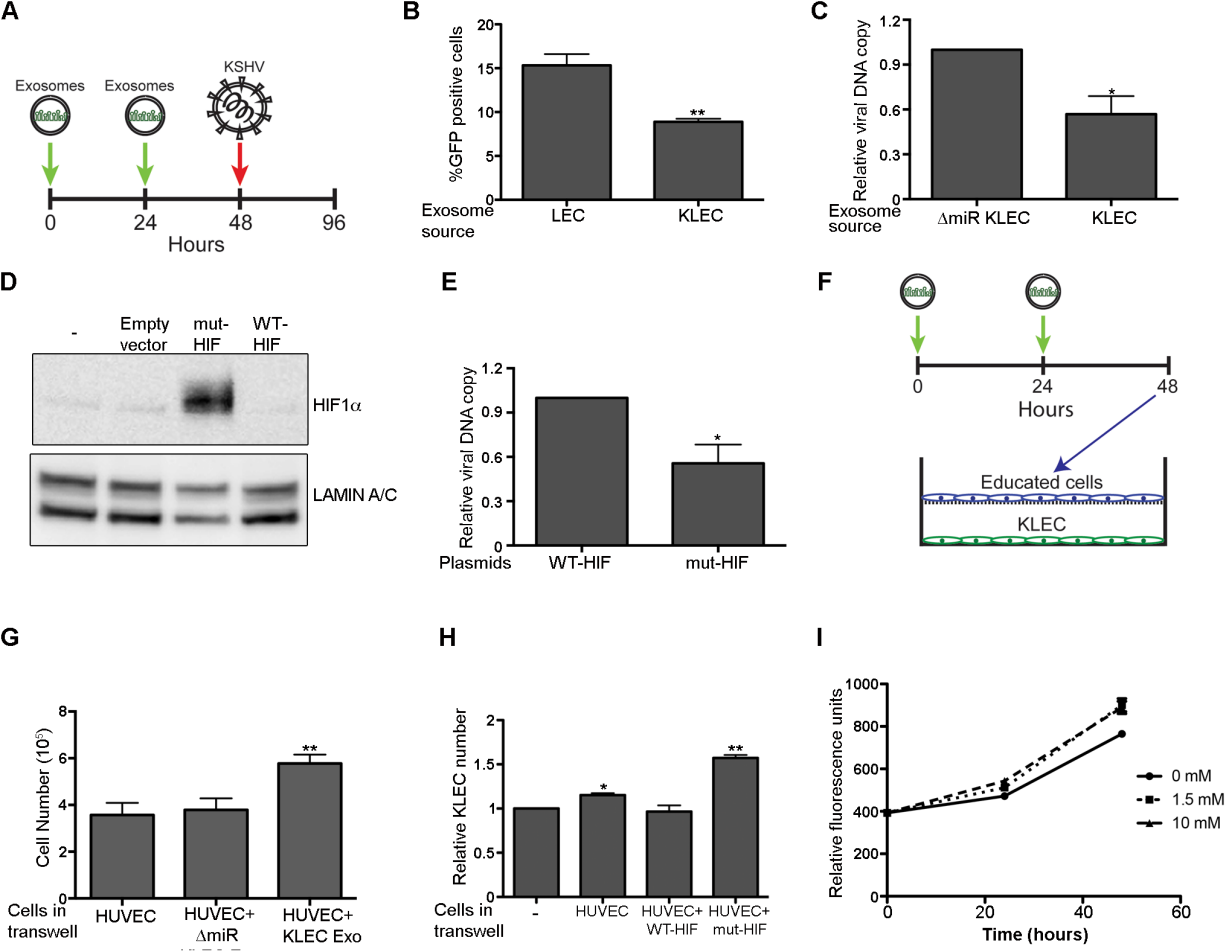
Inducing the reverse Warburg effect inhibits KSHV infection and supports KLEC growth. **(A)** Schematic illustration of the procedure used in this study to infect educated cells. 10^5^ non-infected cells were incubated for total of 48 hours when 2.5x10^9^ exosomes were added every 24 hours, and infected with BAC16-derived WT KSHV. Cells were analysed 48 hours’ post KSHV infection. **(B)** Percentage of infected cells after education using LEC or KLEC exosomes. Cells were analysed using flow cytometry. **(C)** KSHV genome copy number in LEC educated using KLEC and ΔmiR-KLEC exosomes, 48 hours post KSHV infection. qPCR was carried out as previously described [61]. **(D)** Expression of HIF1α in LEC expressing the HIF1α P402A/P564A mutant. Lysates from cell infected with empty vector (EV), mutant or WT HIF1α were separated by SDS/PAGE and analysed by immunoblot for expression of HIF1α. **(E)** LEC expressing WT and mut-HIF1α were infected with BAC16-derived WT KSHV and examined for KSHV genome copy number using qPCR. **(F)** Schematic illustration of the procedure used in this study to co-culture KLEC with educated cells. 10^5^ HUVEC cells were incubated for total of 48 hours when 2.5x10^9^ exosomes were added every 24 hours. 10^5^ of the educated cells were washed, trypsinized and then co-cultured with 10^5^ KLEC in Transwell plates for 2 days. **(G)** KLEC growth in the presence of educated HUVEC. KLEC were trypsinised and each condition was counted using both hemocytometer and the Millipore Scepter 2.0 Handheld Automated Cell Counter. **(H)** KLEC growth in the presence of HUVEC over-expressing HIF1α. The indicated cells were co-cultured and counted as described in (G). **(I)** KLEC media was supplemented with 1.5 or 10mM sodium L-Lactate. Cell number was measured using the CellTiter-Fluor assay (promega) after 24 and 48 hours. All bar graphs present the mean and standard deviation of 3 biological repeats. Statistical significance was denoted by *P < .05, **P < .01, ***P < .001.

How might metabolic transformation of the microenvironment enhance the fitness of KSHV? One possibility is that it sensitises surrounding cells to viral infection. To test this, we infected different educated cells with KSHV (Fig 5A). Contrary to our expectations we observed a 50% decrease in infection of cells educated using KLEC-derived exosomes compared to those educated using LEC exosomes (Fig 5B). Similarly, DNA analysis of cell educated using KLEC and ΔmiR-KLEC exosomes showed a similar decrease in the viral copy number in cells educated using KLEC derived exosomes (Fig 5C). To test whether this unexpected effect is due to the metabolic changes induced by these exosomes, we mimicked the exosome metabolic effect by expressing the HIF1α P402A/P564A stable mutant [44] in LEC (Fig 5D). Over-expression of HIF1α had a similar effect to exosome treatment, leading to a 40% reduction in the viral copy number 48 hours’ post KSHV infection (Fig 5E). Thus, it appears that aerobic glycolysis inhibits KSHV infection, and that exosomes secreted from infected cells in fact prevent KSHV spreading into new cells.

### Inducing reverse Warburg effect supports KLEC growth

The fact that uptake of exosomes secreted from LEC reduces viral spreading suggests other benefit for the virus. It has been suggested that the reverse Warburg effect in stromal cells supports growth of cancer cells [3, 4]. We therefore hypothesised that inducing aerobic glycolysis in nearby non-infected cells supports the growth of KSHV-infected cells. To test this hypothesis, we co-cultured KLEC in Transwell plates with HUVEC educated with different exosomes (Fig 5F). We found that growing in the presence of cell per-educated with KLEC derived exosomes, promoted KLEC growth by ~40% compared to growing in the presence of cells educated with exosomes from ΔmiR-KLEC (Fig 5G). HUVEC over-expressing stable mutant HIF1α had a similar effect on KLEC growth (Fig 5H), supporting the notion that this increased growth is indeed due to the reverse Warburg effect.

In cancer models, the reverse Warburg effect is thought to support cancer cell growth by promoting the secretion of energy-rich metabolites such as lactate and pyruvate from non-cancerous neighbours. These metabolites are taken up by the cancer cells and used in the mitochondrial TCA cycle, thereby promoting efficient energy production and higher proliferative capacity [3]. Since we have found that uptake of exosomes secreted from KLEC leads to increased levels of lactate and pyruvate in educated cells (Fig 3H and 3I), we tested whether lactate supports KLEC growth. We found that supplementing the growth medium with Lactate promotes KLEC growth, though to a lesser extent than with HUVEC co-culture (Fig 5I), suggesting that lactate responsible for part of this phenotype. Consistent with this, we found that KSHV infection leads to over expression of the monocarboxylate transporters MCT1 and 2 (S6 Fig), supporting our notion that these cells uptake high energy molecules such as lactate and pyruvate to support their growth.

Taken together, these results suggest a metabolic feedback where KSHV infected cells induce aerobic glycolysis in cells in their microenvironment, and those as a result secrete high energy metabolites that support the KSHV infected cells.

### Exosomes secreted from KSHV infected cells induce angiogenesis and migration of non-infected cells

Kaposi's sarcoma (KS) is a highly-vascularised tumour supporting large amounts of neo-angiogenesis. It has been proposed that KSHV directly induces angiogenesis in a paracrine fashion [45]. Consistent with this KSHV infection of endothelial cells in culture induces a number of host pathways involved in activation of angiogenesis and a number of KSHV genes themselves can induce pathways involved in angiogenesis.

**Fig 6:**
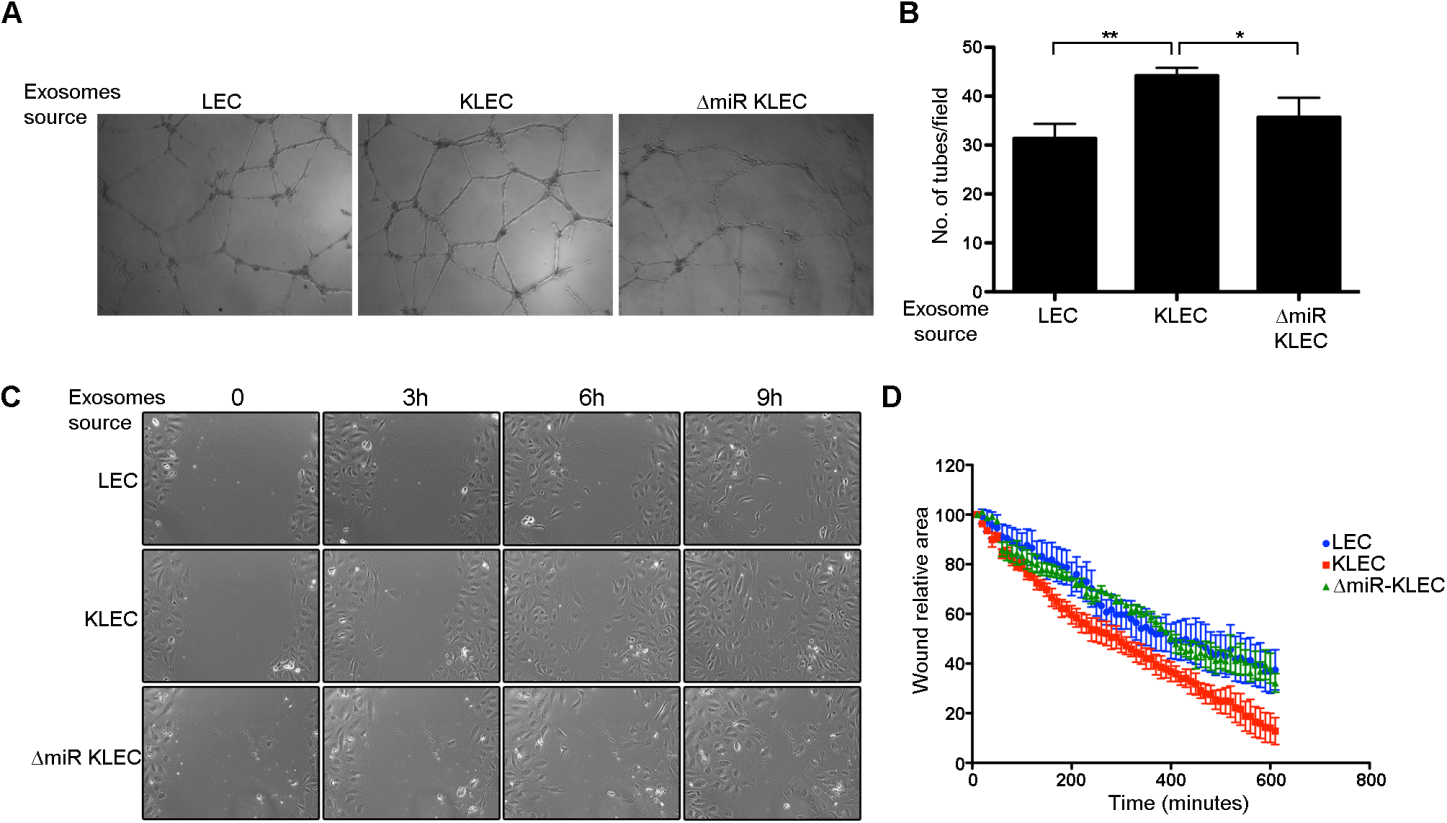
KLEC-derived exosomes increase the angiogenic potential and migration of non-infected endothelial cells. **(A-B)** 10^5^ LEC were educated using 2.5x10^9^ LEC, KLEC or ΔmiR-KLEC—derived exosomes. Angiogenic potential was measured using an endothelial cell tube-formation assay. **(A)** Representative images of tubes formed on matrigel. **(B)** Average number of tubes per field acquired. The graph presents the mean and standard deviation of 3 biological repeats. Statistical significance was denoted by *P < .05, **P < .01, ***P < .001. **(C-D)** Cell migration of educated HUVEC was measured using wound healing assay (scratch assay). Educated HUVEC were seeded at 80% confluence in a 24-well plate and allowed to equilibrate overnight. Once scratched, plates were immediately placed in Nikon Biostation CT or Zeiss Cell Observer and Images were acquired for 16 hours. **(C)** representative images of educated HUVEC at 0, 3, 6 and 9 hours’ post scratch. **(D)** Relative scratch area over time as analysed using ImageJ.

We have previously shown that expression of the KSHV miRNAs leads to stabilisation of HIF1α in infected cells [11]. Here we found that exosomal transfer of KSHV miRNAs leads to similar affect also in non-infected cells (Fig 3G). Since HIF1α is as a master regulator of angiogenesis[46], we hypothesis that KSHV uses exosomes to induce angiogenesis also in non-infected cells. To test this, we determined the angiogenic ability of non-infected LEC using an endothelial cell tube-formation assay. As shown in Fig 6A and 6B, LEC educated by KLEC-derived exosomes have greater angiogenic potential compared to LEC educated by LEC or ΔmiR-KLEC derived exosomes. This suggests that viruses can use exosomes secretion as a mechanism to enrich their growth environment by increasing the angiogenic potential of non-infected endothelial cells.

It was previously shown that exosomes collected from patient blood or KS models can induce migration [13]. In order to directly test if this phenotype is due to transfer of the viral miRNAs we educated HUVEC using exosomes from LEC, KLEC and ΔmiR-KLEC, and tested their migration capability using wound assay. We found that while HUVEC educated using LEC or ΔmiR-KLEC exosomes migrate similarly, HUVEC educated using KLEC derived exosomes migrate faster (Fig 6C and 6D).

Taken together these results suggest that KSHV uses exosomes to induce angiogenesis and migration of non-infected cells around its host cell, and present a potential mechanism allowing the virus to enrich its microenvironment.

## Discussion

Viruses have long served as tools in molecular and cellular biology to study a variety of complex processes. In this study, we reveal a novel mechanism by which oncogenic herpesviruses can regulate the nature of their microenvironment, which has implications for cancer cell biology.

**Fig 7:**
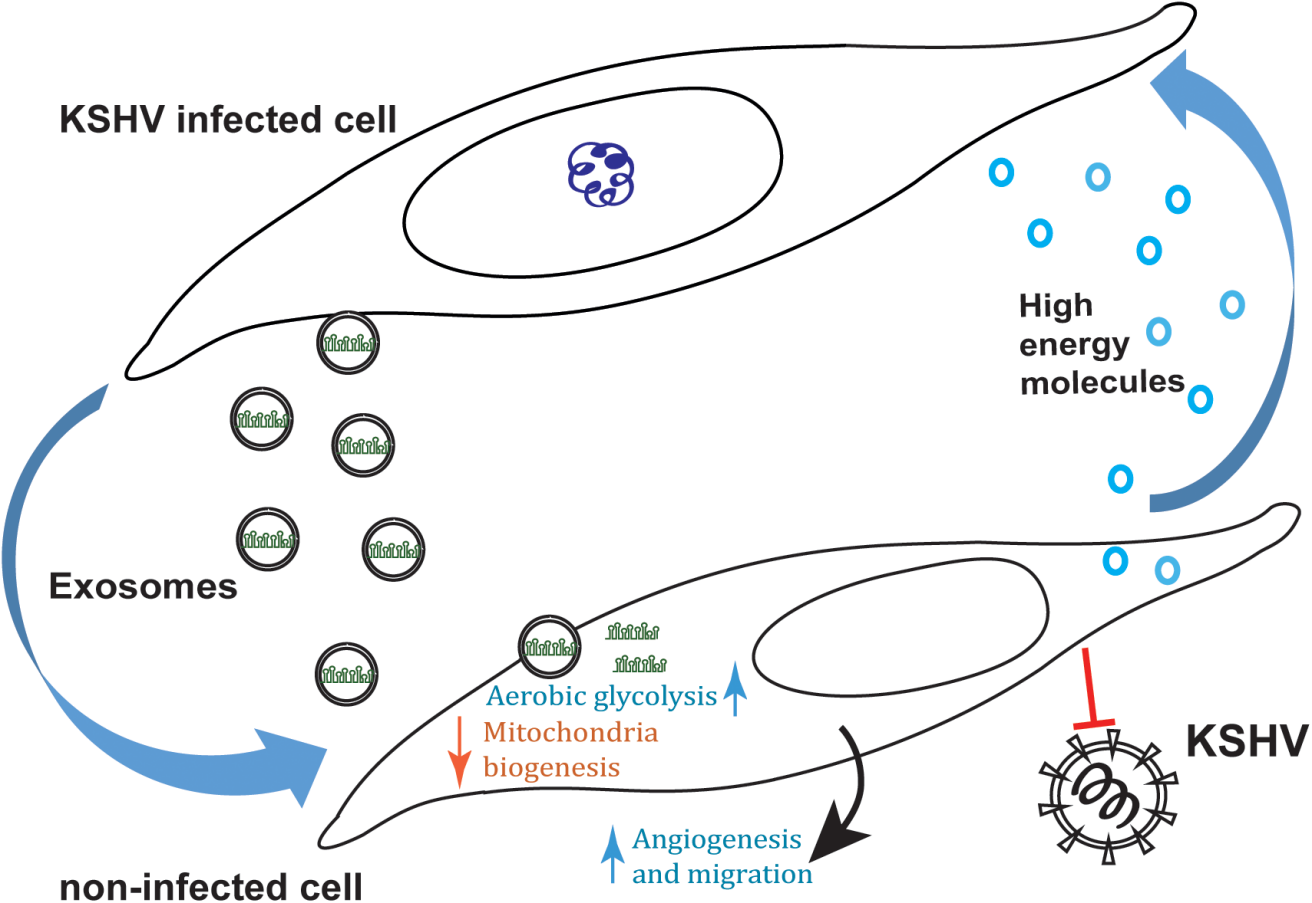
KSHV recruits its microenvironment by transferring its miRNAs in exosomes. Exosomes secreted from latently infected primary LEC transfer the viral miRNAs into neighbouring cells. While these cells remain uninfected, the viral miRNAs are active in them and down regulate expression of their target genes. This results in a metabolic shift toward aerobic glycolysis and to reduced mitochondria biogenesis. This process decreases the sensitivity of the exosome-recipient cells to KSHV but increase their angiogenic potential and supports replication in the parental cells by supplying high energy molecules.

We have found that KSHV not only regulates its host cell metabolism, but also alters the metabolism of neighbouring non-infected cells. We report that exosomes secreted from latently infected primary LEC selectively transfer the viral miRNAs into neighbouring cells. While these cells remain uninfected, the viral miRNAs are active in them and down regulate expression of their target genes. This results in a metabolic shift toward aerobic glycolysis and reduced mitochondria biogenesis. Moreover, our data show that this exosomal transfer of miRNAs transforms the exosome-recipient cells into ‘feeder cells’ producing a microenvironment that is more supportive for host cell growth. We suggest this is due to secretion of high energy molecules, such as lactate and pyruvate, that can be used by the infected cells (Fig 7). Moreover, we found that this flow of genetic information increases the angiogenic potential of non-infected cells and therefore, could further enrich the infected cells microenvironment to support their fitness.

Our data suggest that KSHV uses a miRNAs-based mechanism to manipulate the metabolism of cells in its microenvironment. This is based on: (i) our previous observation that expression of the KSHV miRNAs is sufficient to induce aerobic glycolysis, (ii) our observation that the KSHV miRNAs are active in recipient cells to regulate the same metabolic target genes as in KSHV infected cells and (iii) the fact that exosomes secreted from ΔmiR-KLEC, which do not contain the viral miRNAs, do not have the same effect. Nevertheless, while our data show that the KSHV miRNAs are the driving force behind this metabolic phenotype, the possibility that other components in these exosomes might support it, still needs to be further investigated.

To some extent it is not unexpected that KSHV miRNAs will be present in exosomes secreted from latent cells, since these miRNAs are highly expressed in them. Indeed, it was shown that these miRNAs are present in exosomes collected from plasma of KS patient or KS mouse models [13]. While identification of viral miRNAs in the blood might be useful for diagnostic purposes, it is hard to appreciate the biological advantage for the virus by transferring these miRNAs in the blood stream. Here we show for the first time that the KSHV miRNAs are selectively enriched in exosomes secreted from infected cells. Critically we show the physiological functionality of this exosomal transfer in shaping the metabolic feature of the infected cells microenvironment and the advantage of this local transfer for virally infected cells. Although exosomes secreted from LEC and KLEC contain both viral and cellular miRNAs, our results support the notion that the KSHV miRNAs are the driving force behind this metabolic transformation since it is not induced by exosomes secreted from cell infected with a mutant virus, lacking the miRNAs cluster (ΔmiR-KLEC). Our results also show that although the KSHV miRNAs are highly expressed in infected cells, much lower levels are sufficient to regulate their target genes and to induce aerobic glycolysis in surrounding cells. These results are consistent with other studies showing that exosomes can transfer miRNAs in sufficient levels to regulate their target genes in the recipient cells [19, 47–49]. We therefore suggest that infected cells express the viral miRNAs in much higher levels than those required to regulate their target genes, to ensure their inclusion in the secreted exosomes.

Our results suggest that regulation of cell metabolism by miRNAs transfer is not unique for KSHV but may present a more general mechanism used by other viruses and cancer cells. EBV, a close relative of KSHV, encodes at least 40 miRNAs, many of which regulate the same genes and pathways as the KSHV miRNAs [35]. We show here that EBV can transfer these miRNAs in exosomes and these can also affect mitochondrial respiration in exosome recipient cells. It was previously shown that exosomes secreted from EBV infected cells also transfer proteins that might be involved in altering the metabolism of recipient cells [22]. While our results do not rule out this possibility, the fact that EBV and KSHV are suggested to share many of their miRNAs target genes supports our model that viral miRNAs transfer is the driving force behind this metabolic regulation.

Herpesviruses account for most of the viral encoded miRNAs, though other DNA and even RNA viruses also encode miRNAs [50]. miRNAs are likely to be invisible to the adaptive immune response. Therefore, transferring miRNAs via exosomes would be advantageous during persistent infection since it allows viruses to recruit cells in their vicinity without producing and releasing new viral particles, a process that requires energy and that exposes them to the immune system. Moreover, many cellular miRNAs are also known to be involved in regulation of energy metabolism [38]. miR-210 is highly expressed under hypoxic conditions to alter cellular processes including cell cycle regulation, mitochondria function, apoptosis and angiogenesis [51]. Hypoxia can arise because of oxygen diffusion limitation in avascular primary tumours or due to abnormal tumour microvascularization. For these reasons, these cells might also have poor nutrient supply. Our results suggest the miR-210, which is over-expressed under these conditions, can be transferred by exosomes into cells in the tumour microenvironment. Inducing the Warburg effect in their microenvironment by transferring miR-210 into normoxic non-cancer cells can support the growth of the hypoxic cancer cells by supplying high-energy molecules such as lactate and pyruvate. Our results with miR-210, raise the possibility that other onco-miRs can be transferred into the microenvironment to support the tumour growth.

Our results suggest that altering their host metabolism by miRNA transfer is a novel mechanism used by oncogenic viruses to influence their hosts. One outcome of this, is growth support for the virus host cell. However, cell metabolism has also been shown to be involved in regulation of other cellular processes that are relevant for KS development and prolong infection. Cell metabolism and specifically oxidative metabolism and glycolysis were also shown to regulate both innate and adaptive immune systems [52]. Moreover, it was recently shown that glucose consumption by tumours metabolically restricts T cells, thereby allowing tumour progression [53]. Our results suggest that viruses might use exosomes to create a hypoglycaemic microenvironment that similarly suppress the immune response against infected cells. Thus, our results raise the possibility that viruses use exosomes to shape the metabolism of their microenvironment during persistent infection, as a mechanism to evade the immune system.

It was previously shown that exosomes secreted from breast cancer cells can inhibit glycolysis in the pre-metastatic niche [19] and that exosomes secreted from cancer associated fibroblast can induce aerobic glycolysis in nearby cancer cells [18]. Here we show the opposite effect, whereby uptake of exosomes from herpes viruses-infected cells induce aerobic glycolysis in surrounding normal cells. This is the first-time exosomes are shown to induce the reverse Warburg effect and presents a new mechanism by which cancer cells can recruit cells in their microenvironment to support their growth.

KSHV miRNAs were shown to regulate many other cellular processes such as cytokine responses, immune recognition, cell survival, transcriptional reprogramming and angiogenesis [54–56]. Therefore, transfer of the viral miRNAs via exosomes may alter a wide spectrum of processes, adding to the complexity of the relationship between viruses and their hosts. The cross talk between KS cells and their microenvironment warrants further study, with a view to identifying novel therapeutic targets.

We present a novel mechanism allowing viruses to regulate the metabolism and migration of cells in their vicinity in a way that supports their fitness by transferring their genetic material via exosomes. We suggest that viruses and cancer cells use this mechanism to shape a specific metabolic niche that will favour their proliferation. It also implies that, similar to cancer cells, latently infected cells depend on their environments for sustained growth. Targeting this miRNA transfer-based metabolic cross-talk between diseased cells and their microenvironment could therefore open a new therapeutic window.

## Materials and methods

### Cell culture

HUVEC and LEC were purchased from Promocell and grown in endothelial growth medium 2 and MV2 (Promocell) respectively. Both cell types were used for experiments before passage 8. To exclude exosomes derived from the FBS, it was subjected to centrifugation of 120,000g for 3 hours. iSLK producing cells were kindly provided by Rolf Renne (University of Florida). KSHV producing iSLK cells were cultured in DMEM (Invitrogen), supplemented with 10% FBS, 1μg/ml Puromycin, 250μg/ml Geneticin and 1200μg/ml Hygromycin. HEK293T (ATCC) and HCT116 (ATCC) cells were cultured in DMEM (Invitrogen), supplemented with 10% FBS. EBV producer line Akata (kindly provided by Paul Farrell, Imperial College) and EBV negative Akata (kindly provided by Andrew Bell, University of Birmingham) were cultured in RPMI 1640 (Invitrogen) supplemented with 10% FCS.

### KSHV preparation and establishment of stable infections

Wild type and ΔmiR-cluster KSHV were prepared from iSLK cells as previously described [23]. Early passage LEC were infected and selected using 50ug/ml Hygromycin B (Invitrogen). Cells were tested for 100% infection (GFP positive) before carrying on any experiment. Repeats for each experiment were performed using different KSHV infections.

### Exosomes purification

Uninfected LEC and LEC infected with WT or ΔmiR-cluster were grown up to approximately 80% confluency. Infected cells were cultured for at least 6 days before collecting media to verify latency establishment, and were grown up to passage 8. Medium was replaced every 48, and kept at 4°C for up to 7 days. The medium was subjected to centrifugation of 300g for 5 minutes, 2000g for 10 minutes and concentrated with a Centricon Plus-70 filter (Millipore) according to the manufacturer’s instructions. The media was then subjected to centrifugation of 10,000g for 1 hour, and was filtered using 0.22μM filters. To purify exosomes, the sample was subjected to ultracentrifugation at 120,000xg for 1 hour and washed once with PBS. Exosomes pellet was resuspended in PBS, quantified for protein concentration and particle number, and stored at -80°C.

### Negative staining for TEM

The exosome suspensions in PBS were incubated on formvar- and 2 nm carbon-coated copper grids overnight at 4°C in a humidified chamber. They were then washed twice in ddH20 by dipping onto the surface of a water droplet and then stained with 2% aqueous uranyl acetate for 2.5 minutes. The stain was drawn off with cartridge paper to leave a thin negative stain. The sections were examined in a Jeol 1010 microscope. Images were taken with a Gatan Orius SC100B charge-coupled device camera and analysed with Gatan Digital Micrograph.

### Vesicles characterization by Nanosight NS300

Characterisation of vesicle size distribution and concentration was performed using Nanoparticle Tracking Analysis (NTA) (Malvern Instruments, Nanosight NS300). Sample size distributions were calibrated in a liquid suspension by the analysis of Brownian motion via light scattering. Nanosight provides single particle size and concentration measurements.

### Small RNA sequencing and analysis

Total RNA was extracted from exosomes or from their respective parental cells (3 biological replicates for each condition) using the miRNeasy mini-kit (Qiagen) and was quantified using Qubit RNA HS assay kit. 200ng or 500ng of RNA from exosomes or cells respectively, were used for small RNA libraries using the NEBNext Multiplex Small RNA Library Prep Set for Illumina (NEB) according to the manufacturer’s instructions. Constructed libraries were assessed using the BioAnalyzer 2100 (Agilent) and KAPA Library Quantification Kit (KAPA Biosystems). Quantified libraries were then sequenced using the single-end sequencing protocol at 36bp.

Expression of human and KSHV miRNA reads were quantified using PaTMaN [57] rapid aligner to the miRbase release 21 database of miRNA sequences [http://mirbase.org] allowing for 1 mismatch (or counting only perfect matches if more than one miRNA was matched). Normalisation and differential expression were performed using the DESeq2 method [58], whilst multiple testing correction employed independent hypothesis weighting (IHW) for greater statistical power [59].

### Exosomes treatment and labelling

For education experiments 10^5^ non-infected cells were incubated for total of 48 hours when 2.5x10^9^ exosomes were added every 24 hours. For biological replicates of all experiments, we used exosomes from separate KSHV infections. For Transwell co-culture experiments, indicated cells were grown on both compartments for total of 5 days and media was replaced every other day. For exosome-tracking experiments, purified exosomes were fluorescently labelled using Carboxyfluorescein succinimidyl diacetate ester (System Biosciences). Labelled exosomes were washed in 20 ml of PBS, collected by ultracentrifugation, and resuspended in PBS.

### Basal cellular respiration rate

Cells were seeded in XF 24-well cell culture microplates (Seahorse Bioscience) at 4 × 104 cells/well (0.32 cm^2^) in 200μl growth medium and then incubated at 37°C/5% CO2 for 20–24 hours. Assays were initiated by removing the growth medium from each well and replacing it with 600μl of assay medium pre-warmed to 37°C. The cells were incubated at 37°C for 30 minutes to allow media temperature and pH to reach equilibrium before the first-rate measurement. Prior to each rate measurement, the XF24 Analyzer gently mixed the assay media in each well for 3 min to allow the oxygen partial pressure to reach equilibrium. Following mixing, OCR and ECAR were measured simultaneously for 4 min to establish a baseline rate. The assay medium was then gently mixed again for 3 min between each rate measurement to restore normal oxygen tension and pH in the microenvironment surrounding the cells. Uncoupled, maximal and non-mitochondrial respiration was determined after the addition of 5 μM oligomycin, 1 μM carbonyl cyanide 4-(trifluoromethoxy)phenylhydrazone (FCCP) and 2 μM antimycin-A. All chemicals were from Sigma-Aldrich.

### Glucose uptake

Glucose uptake was measured by incubating cells with 30μM glucose analogue 6-NBDG (Invitrogen) for 15 minutes. Cells where then washed and trypsinized and their fluorescence (λex: 465 nm, λem: 540nm) was measured by flow cytometry.

### Mitochondrial structure and volume

Cells were loaded with 5μM Calcein-AM and 50nM MitoTracker Red (Invitrogen; 37 °C, 30 minutes) in growth media for 30 minutes and Z-series of images were acquired using a Zeiss LSM 510 system (Carl Zeiss, Inc., Cambridge, UK), as previously described [11]. Maximal projection of images was used to quantify the area of green (Calcein) and red (mitoTracker Red) signal. Mitochondrial area was defined relative to cytoplasmic area as ‘area red/area green’. Images were analyzed using the MetaMorph Microscopy Automation & Image Analysis Software (Molecular Devices). The two channels (Calcein-AM and MitoTracker Red) were separated and threshold in order to acquire two separate binary images. To ensure reproducibility, for each biological repeat, we analysed images of at least 10 fields, when each field contain between 5–10 cells.

### Metabolites extraction

Metabolites were extracted according to the Human Metabolome Technologies, Inc. protocol. Shortly, 2x10^6^ cells were washed twice using 5% mannitol solution and metabolites were extracted by adding methanol for 30 second. The extracted solution was centrifuged 2300g at 4°C for 5 minutes, filtered and evaporated using centrifugal evaporator. Analysing the ionic metabolites including in these cells by CE-TOFMS and CE-QqQMS was performed by Human Metabolome Technologies, Inc. The results in the manuscript present 3 biological repeats, where cells were educated using exosomes from different KSHV infections.

### Western blotting and antibodies

Cells were lysed in RIPA buffer (300mM Sodium Chloride, 1% NP-40, 0.5% Sodium deoxycholate, 0.1% Sodium dodecyl sulphate and 50mM Tris pH 8.0). Exosomes were lysed directly into 1x Laemmli sample buffer (Bio-Rad Laboratories). Equal amounts of protein were resolved on Mini-PROTEAN TGX Precast gels (Bio-Rad Laboratories). Antibodies against CD63 (Invitrogen), α-TUBULIN (Sigma-Aldrich), HIF1α (BD Transduction Laboratories), LAMIN A/C (Santa Cruz Biotechnology), EBV-GP125 (GeneTex) and KSHV-ORF8 (ThermoFisher Scientific) were detected with IRDye secondary antibodies (LI-COR) or HRP-conjugated secondary antibodies. Images were acquired using the Li-COR Odyssey Imaging System or the GE Healthcare Imagequant LAS 4000.

### qPCR and qRT–PCR

Genomic DNA for qPCR was extracted using the QIAamp DNA mini-kit (Qiagen). Cell total RNA was extracted using either the RNeasy mini-kit or the miRNeasy mini-kit (Qiagen). Exosomal RNA was extracted using the miRNeasy mini-kit (Qiagen). KSHV genome copy numbers were quantified by qPCR as previously described [11].

cDNA synthesis for qRT–PCR quantification of mature miRNAs was performed using the Exiqon Universal cDNA Synthesis Kit II according to the manufacturer's instructions. Detection of the mature KSHV miRNAs was performed using the KSHV-miR LNA PCR primer sets (Exiqon). Where indicated cellular small nucleolar RNA RNU66 or S5 rRNA were used as a reference RNA. Importantly, the KSHV LNA PCR primer sets do not give any background detection for negative control (such as non-infected cells or ΔmiR-KLEC).

### 3’UTR Luciferase reporter assay

The reporter plasmids for the 3’UTRs of the indicated genes were previously described [11]. Cells expressing each of the reporter plasmids were incubated for 48 hours with the indicated exosomes for 48 hours. Cells were harvested according to the Dual-Luciferase Reporter assay system (Promega). Luciferase activity was measured using a Fluoroskan Ascent FL luminometer (ThermoScientific). Firefly activity was normalised to internal Renilla luciferase levels.

### Endothelial tube formation assay

Growth factor–reduced Matrigel (Becton Dickinson) was placed in 96-well tissue culture plates (75μl/well) and allowed to gel at 37°C for 30 minutes. Then 1x10^4^ LEC, pre-incubated with exosomes derived from LEC, KLEC or ΔmiR-KLEC, were added to each well and incubated at 37°C for 24 hours. Morphological changes were visualised using phase contrast microscope.

### Wound healing assay

Educated HUVEC were seeded in 12 well plates and grown overnight to confluence. A 200μl tip was used to make a straight scratch and plates were immediately placed in Nikon Biostation CT or Zeiss Cell Observer. Images were acquired for 16 hours and analysed for scratch area using ImageJ.

### Overexpression of miR-210

miR-210 was cloned using the Gateway Cloning protocol (Invitrogen). Shortly, the mature microRNA was amplified using the primers:

Forward–GCGGCCGCTGGTACCCTGGACACACAAGGAAA.
Reverse–AGATCTAGGGATCCCAGGTTGGCG.

The PCR product subcloned into the gateway entry vector pENTR/pTER+ [60]. The miRNA was further cloned into the 3rd gen lentiviral promoter-less Gateway destination vector pLenti X1 Puro DEST using the Gateway LR Clonase II enzyme mix (Invitrogen).

## Supporting information

S1 Fig Characterisation of exosomes secreted from KLEC. **(A)** Representatives images of KLEC after selection. **(B)** KSHV genome copy number in KLEC and ΔmiR-KLEC. qPCR was carried out as previously described [61]. **(C)** Relative mRNA levels of LANA and vcyclin in KLEC and ΔmiR-KLEC. mRNA levels were determined by qRT-PCR. TUBB levels were used for normalisation. **(D)** Schematic illustration of the procedure used in this study to extract exosomes from LEC and KLEC. **(E-F)** Exosomes were collected from LEC, KLEC and ΔmiR-KLEC growth medium and analysed by Nanosight NS300 (Malvern) for size distribution (E) and particle concentration (F). **(G)** Lysates from purified exosomes were separated by SDS/PAGE and analysed by immunoblot for expression of the exosomal marker CD9 and Alix. **(H-I)** Expression of the mature KSHV miRNAs in exosomes purified from KLEC and ΔmiR-KLEC growth media. Detection of the mature KSHV miRNAs was performed using the KSHV-miR LNA PCR primer sets (Exiqon). In all panels, except to panel B, the graphs present the mean and standard deviation of 3 biological repeats.
(TIF)

S2 Fig KLEC-derived exosomes are being taken up by naïve cells. LEC were incubated with fluorescently labelled exosomes and analysed using a fluorescence-activated cell sorter (FACS).
(TIF)

S3 Fig KLEC-derived exosomes induce the reverse Warburg effect. **(A)** LEC were educated using the indicated number of exosomes collected from KLEC growth media and analysed using the Seahorse XF24 Analyser for oxygen consumption rate. The bar graph presents the average base line oxygen consumption rate. **(B)** Oxygen consumption rate of uneducated LEC, and LEC and KLEC co-cultured in transwell plates. **(C)** The indicated metabolites concentrations as measured in educated cells using CE-TOFMS and CE-QqQMS (Human Metabolome Technologies, Inc.). **(D)** LEC were educated using KLEC-derived exosomes, then grown for additional 5 days in exosome free media and analysed using the Seahorse XF24 Analyser for oxygen consumption rate. **(E-F)** HUVEC were educated using the indicated exosomes and analysed for oxygen consumption rate using the Seahorse XF24 Analyser (E) or for mitochondria volume (F) as previously described [11]. The bar graph presents the average mitochondrial volume in cells (Mean+SD, n = 3).
(TIF)

S4 Fig Characterisation of exosomes secreted from AKATA cells. **(A)** Lysates from purified exosomes or EBV (10μg) were separated by SDS/PAGE and analysed by immunoblot for the viral protein gp125. **(B)** Lysates from purified exosomes or EBV (10μg) were separated by SDS/PAGE and analysed by immunoblot for expression of the exosomal marker ALIX.
(TIF)

S5 Fig miR-210 is transfer in exosomes to induce reverse Warburg effect. **(A)** Levels of miR210 in exosomes secreted from 293T or HCT-116 force expressing miR210. Detection of mature hsa-miR-210 was performed using a specific LNA PCR primer set (Exiqon). **(B)** Expression levels of ISCU1 in cells educated using miR-210 exosomes. mRNA levels were determined by quantitative real-time PCR (qRT-PCR). Tubulin beta (TUBB) levels were used for normalisation. **(C)** Oxygen consumption rate (OCR) as measured using the Seahorse XF24 Analyser. Cells were seeded at a density of 4x104 cells per well and the assay was performed according to the manufacturer’s Mito stress protocol.
(TIF)

S6 Fig KLEC over express the monocarboxylate transporters MCT 1 and 2. mRNA levels were determined by quantitative real-time PCR (qRT-PCR). Tubulin beta (TUBB) levels were used for normalisation.
(TIF)

S1 Table Expression levels of the KSHV miRNAs in KLEC and KLEC-derived exosomes. The expression level was calculated as fraction of total reads detected in KLEC and KLEC-derived exosomes.
(TIF)

S2 Table Relative expression levels of selected miRNAs in KLEC compared to LEC. (TIF)

S3 Table Relative expression levels of selected miRNAs in KLEC derived exosomes compared to LEC derived exosomes. (TIF)
