# Supplementary figures and images for: Herpesviruses shape tumour microenvironment through exosomal transfer of viral microRNAs
Source: PLoS Pathog. 2017 Aug 24;13(8):e1006524. doi: 10.1371/journal.ppat.1006524 (PMC5570218; doi:10.1371/journal.ppat.1006524)

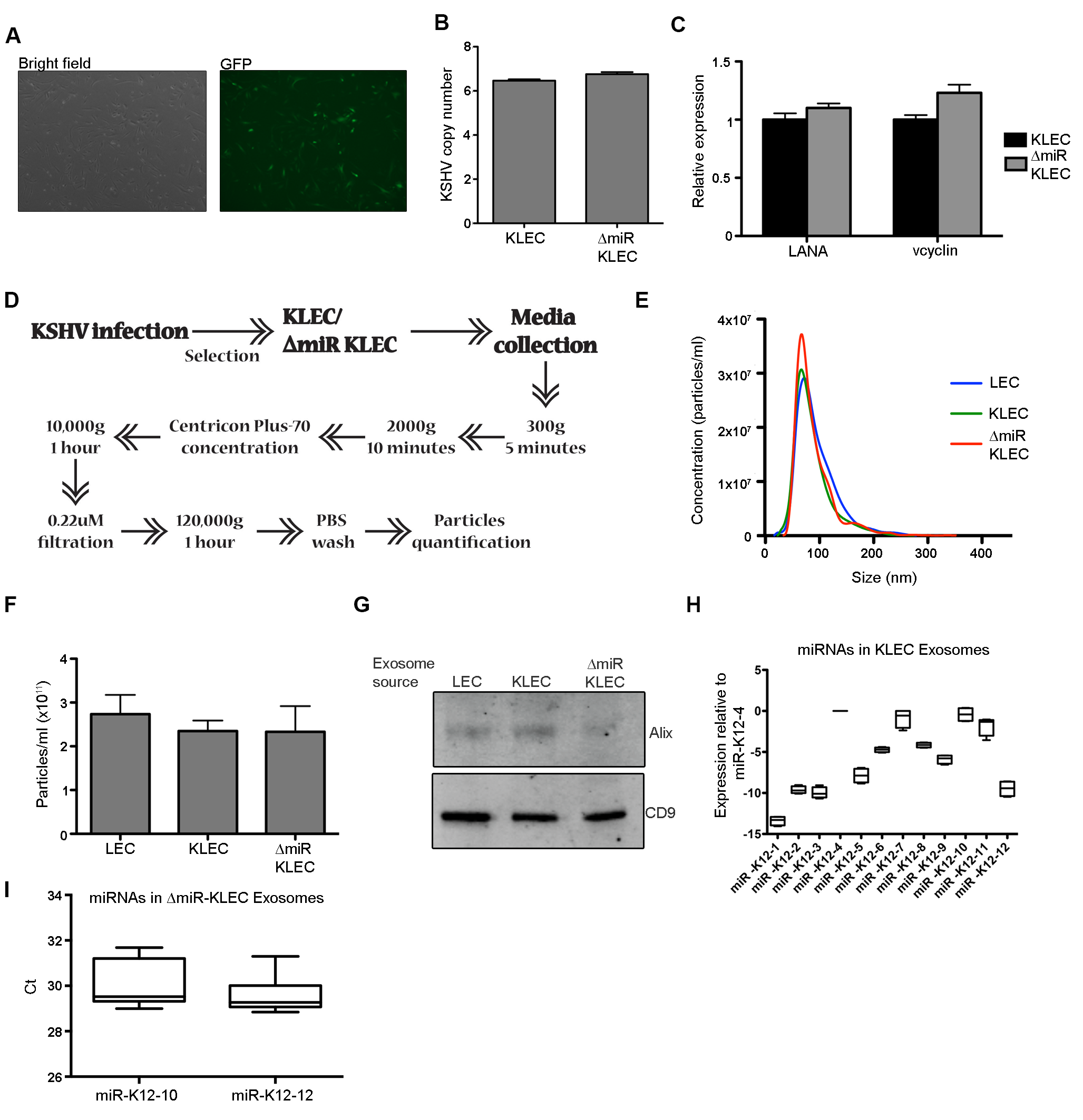

Supplement: S1 Fig — (A) Representatives images of KLEC after selection. (B) KSHV genome copy number in KLEC and ΔmiR-KLEC. qPCR was carried out as previously described [61]. (C) Relative mRNA levels of LANA and vcyclin in KLEC and ΔmiR-KLEC. mRNA levels were determined by qRT-PCR. TUBB levels were used for normalisation. (D) Schematic illustration of the procedure used in this study to extract exosomes from LEC and KLEC. (E-F) Exosomes were collected from LEC, KLEC and ΔmiR-KLEC growth medium and analysed by Nanosight NS300 (Malvern) for size distribution (E) and particle concentration (F). (G) Lysates from purified exosomes were separated by SDS/PAGE and analysed by immunoblot for expression of the exosomal marker CD9 and Alix. (H-I) Expression of the mature KSHV miRNAs in exosomes purified from KLEC and ΔmiR-KLEC growth media. Detection of the mature KSHV miRNAs was performed using the KSHV-miR LNA PCR primer sets (Exiqon). In all panels, except to panel B, the graphs present the mean and standard deviation of 3 biological repeats. (TIF) [file ppat.1006524.s001.tif]

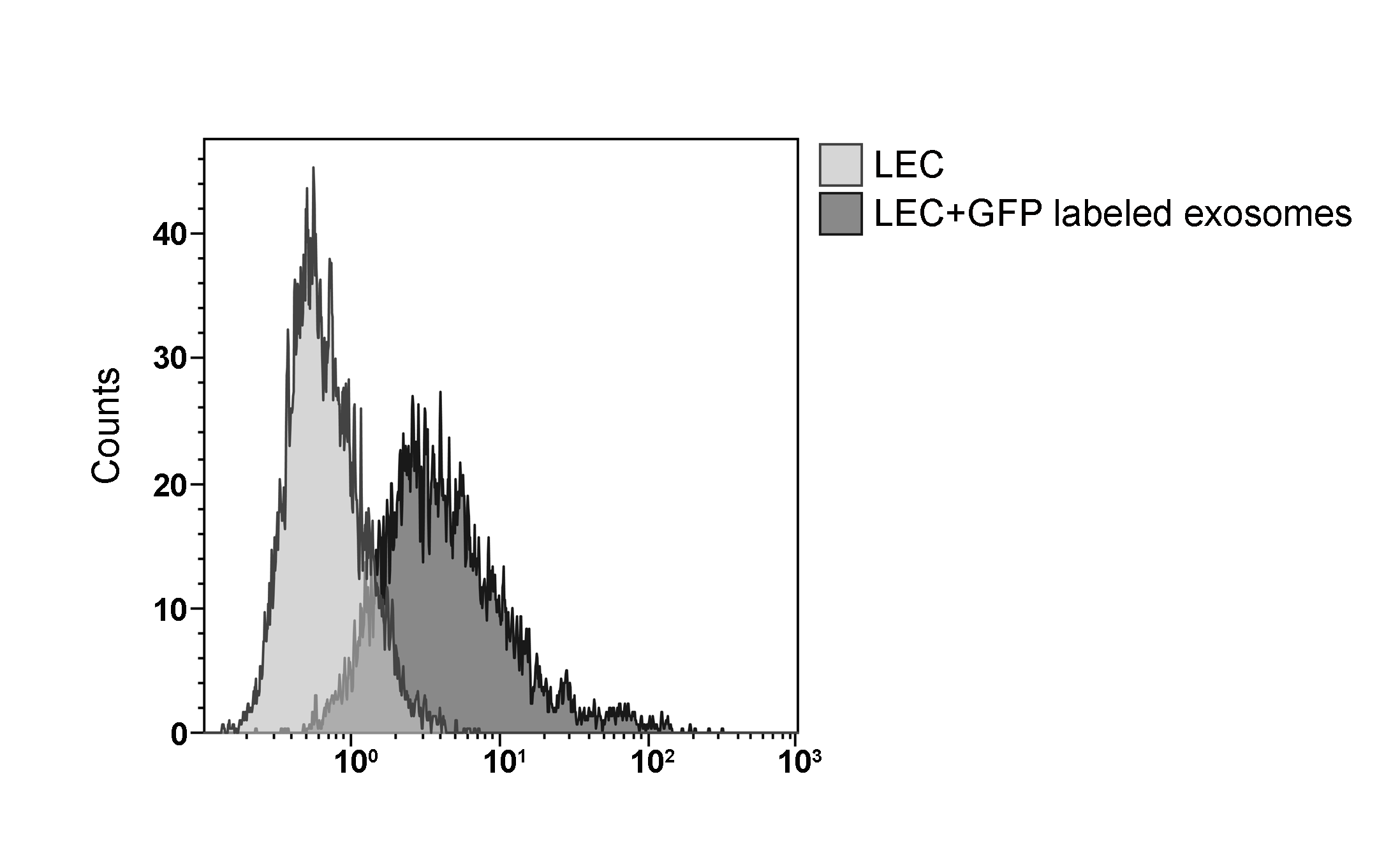

Supplement: S2 Fig — LEC were incubated with fluorescently labelled exosomes and analysed using a fluorescence-activated cell sorter (FACS). (TIF) [file ppat.1006524.s002.tif]

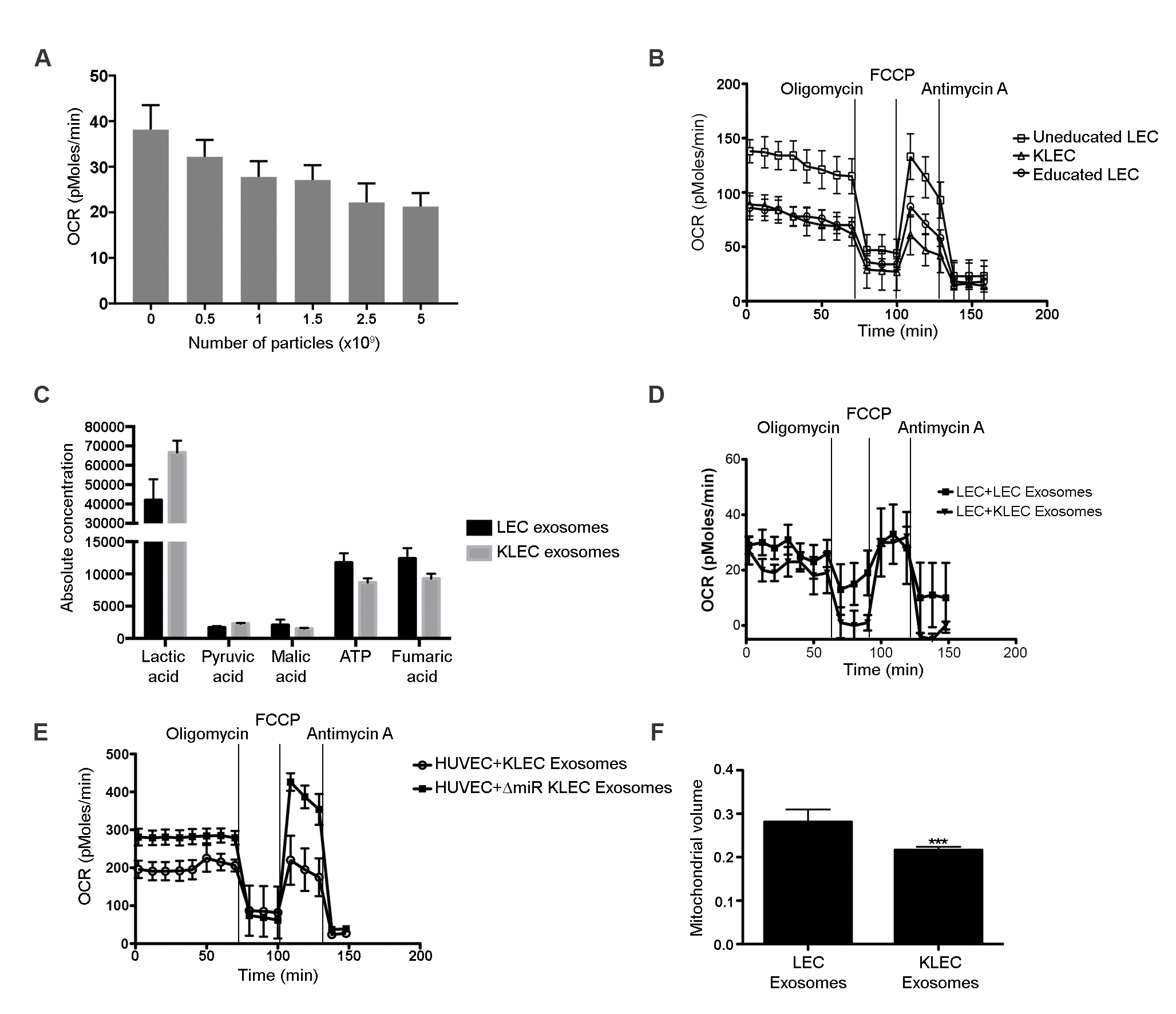

Supplement: S3 Fig — (A) LEC were educated using the indicated number of exosomes collected from KLEC growth media and analysed using the Seahorse XF24 Analyser for oxygen consumption rate. The bar graph presents the average base line oxygen consumption rate. (B) Oxygen consumption rate of uneducated LEC, and LEC and KLEC co-cultured in transwell plates. (C) The indicated metabolites concentrations as measured in educated cells using CE-TOFMS and CE-QqQMS (Human Metabolome Technologies, Inc.). (D) LEC were educated using KLEC-derived exosomes, then grown for additional 5 days in exosome free media and analysed using the Seahorse XF24 Analyser for oxygen consumption rate. (E-F) HUVEC were educated using the indicated exosomes and analysed for oxygen consumption rate using the Seahorse XF24 Analyser (E) or for mitochondria volume (F) as previously described [11]. The bar graph presents the average mitochondrial volume in cells (Mean+SD, n = 3). (TIF) [file ppat.1006524.s003.tif]

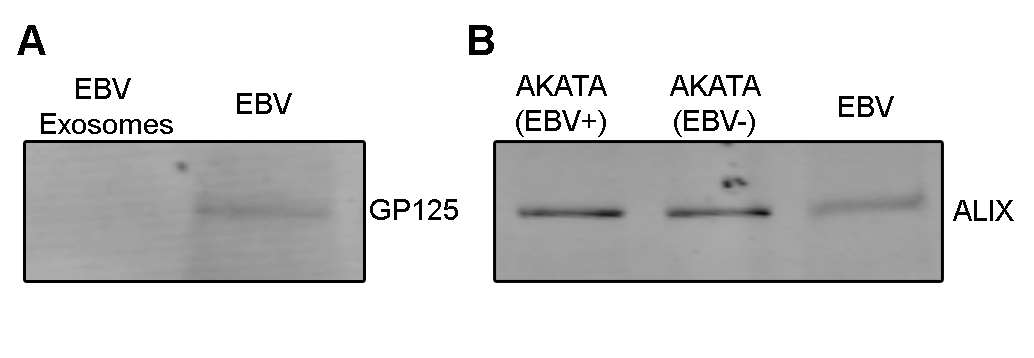

Supplement: S4 Fig — (A) Lysates from purified exosomes or EBV (10μg) were separated by SDS/PAGE and analysed by immunoblot for the viral protein gp125. (B) Lysates from purified exosomes or EBV (10μg) were separated by SDS/PAGE and analysed by immunoblot for expression of the exosomal marker ALIX. (TIF) [file ppat.1006524.s004.tif]

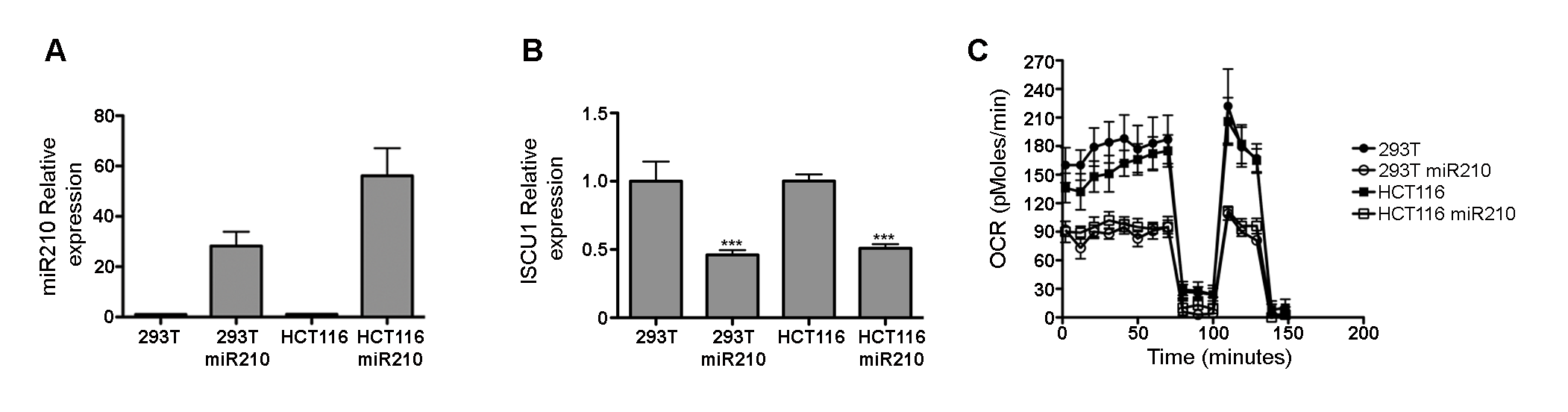

Supplement: S5 Fig — (A) Levels of miR210 in exosomes secreted from 293T or HCT-116 force expressing miR210. Detection of mature hsa-miR-210 was performed using a specific LNA PCR primer set (Exiqon). (B) Expression levels of ISCU1 in cells educated using miR-210 exosomes. mRNA levels were determined by quantitative real-time PCR (qRT-PCR). Tubulin beta (TUBB) levels were used for normalisation. (C) Oxygen consumption rate (OCR) as measured using the Seahorse XF24 Analyser. Cells were seeded at a density of 4x10^4^ cells per well and the assay was performed according to the manufacturer’s Mito stress protocol. (TIF) [file ppat.1006524.s005.tif]

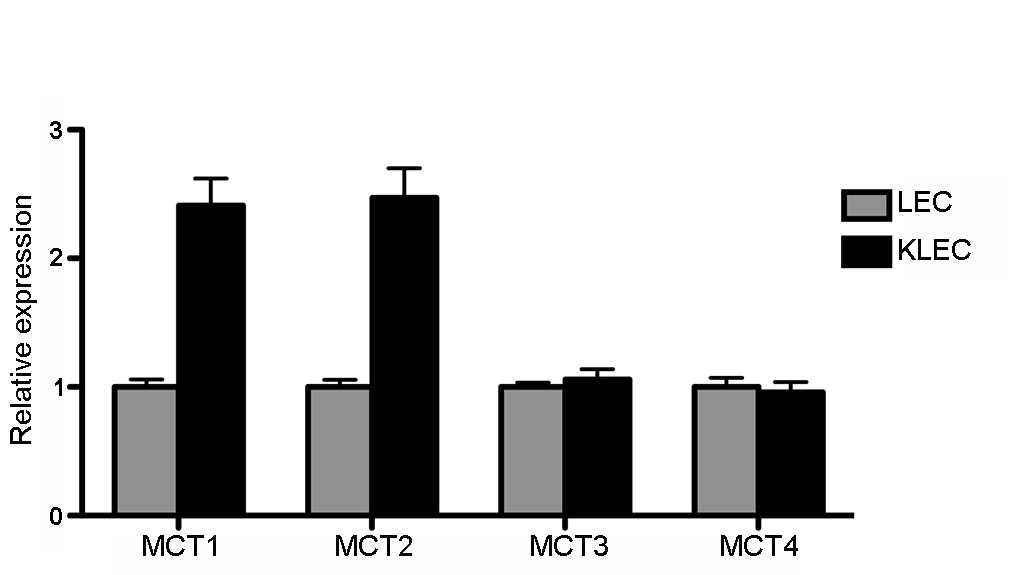

Supplement: S6 Fig — mRNA levels were determined by quantitative real-time PCR (qRT-PCR). Tubulin beta (TUBB) levels were used for normalisation. (TIF) [file ppat.1006524.s006.tif]

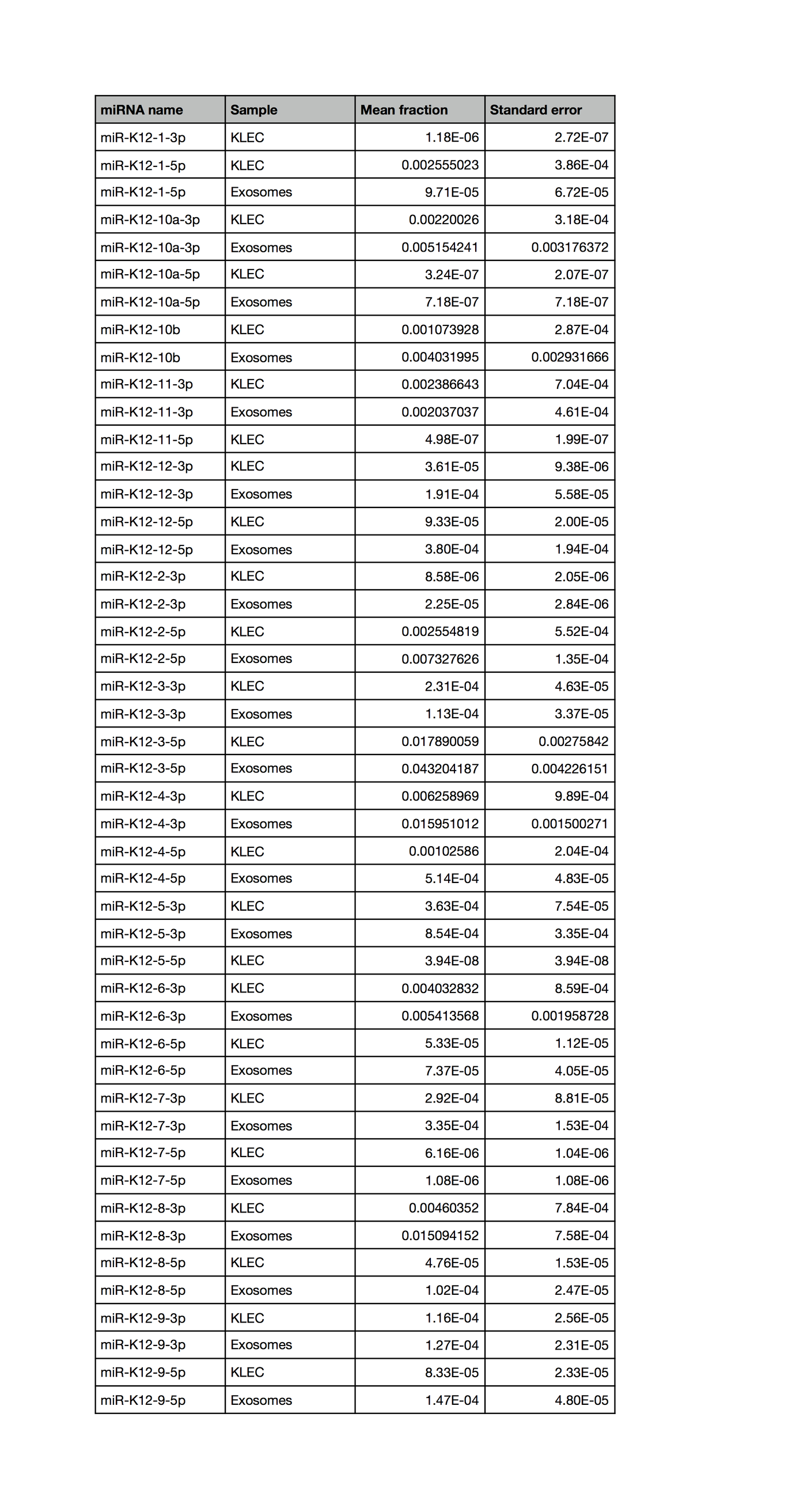

Supplement: S1 Table — The expression level was calculated as fraction of total reads detected in KLEC and KLEC-derived exosomes. (TIF) [file ppat.1006524.s007.tif]

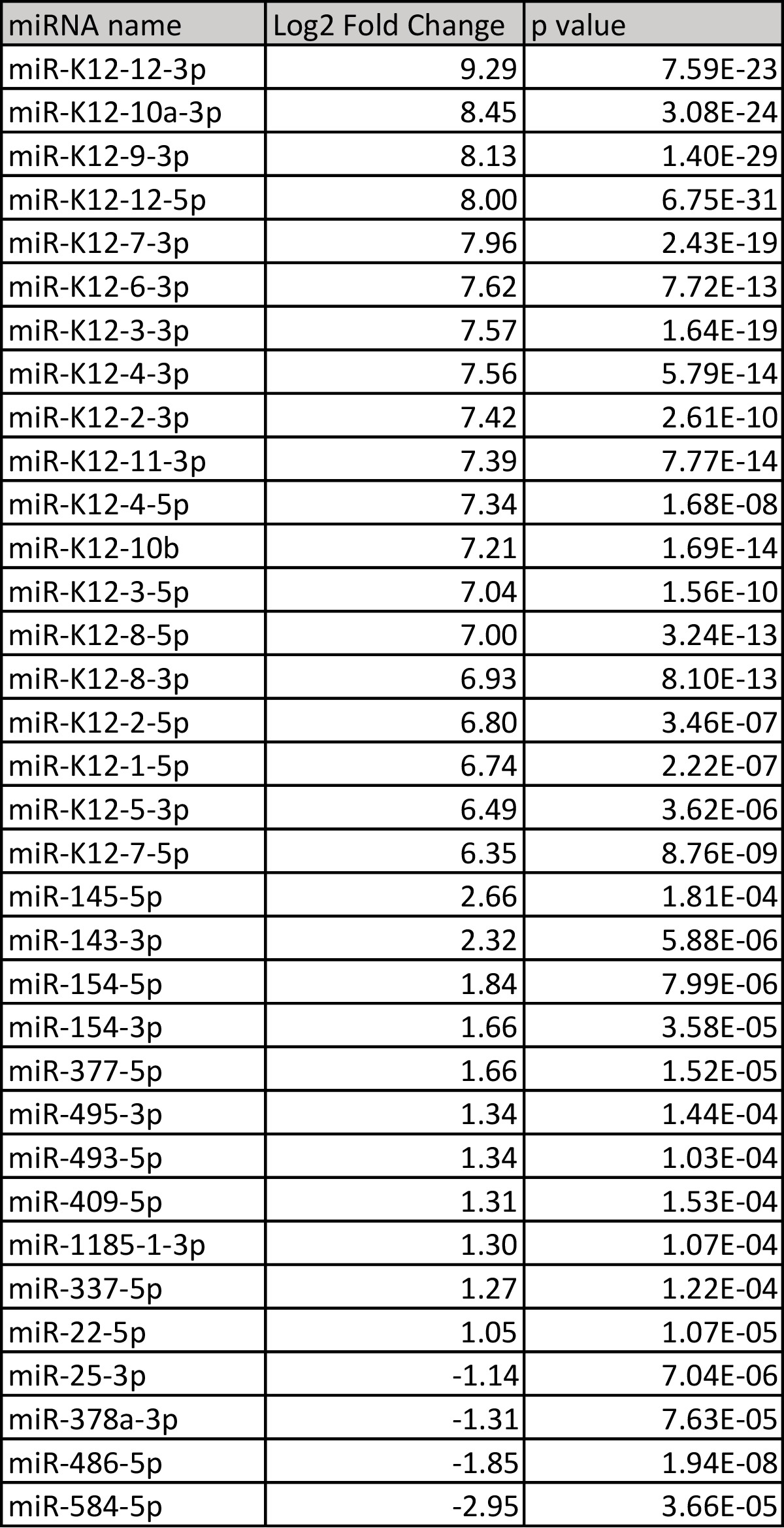

Supplement: S2 Table — (TIF) [file ppat.1006524.s008.tif]

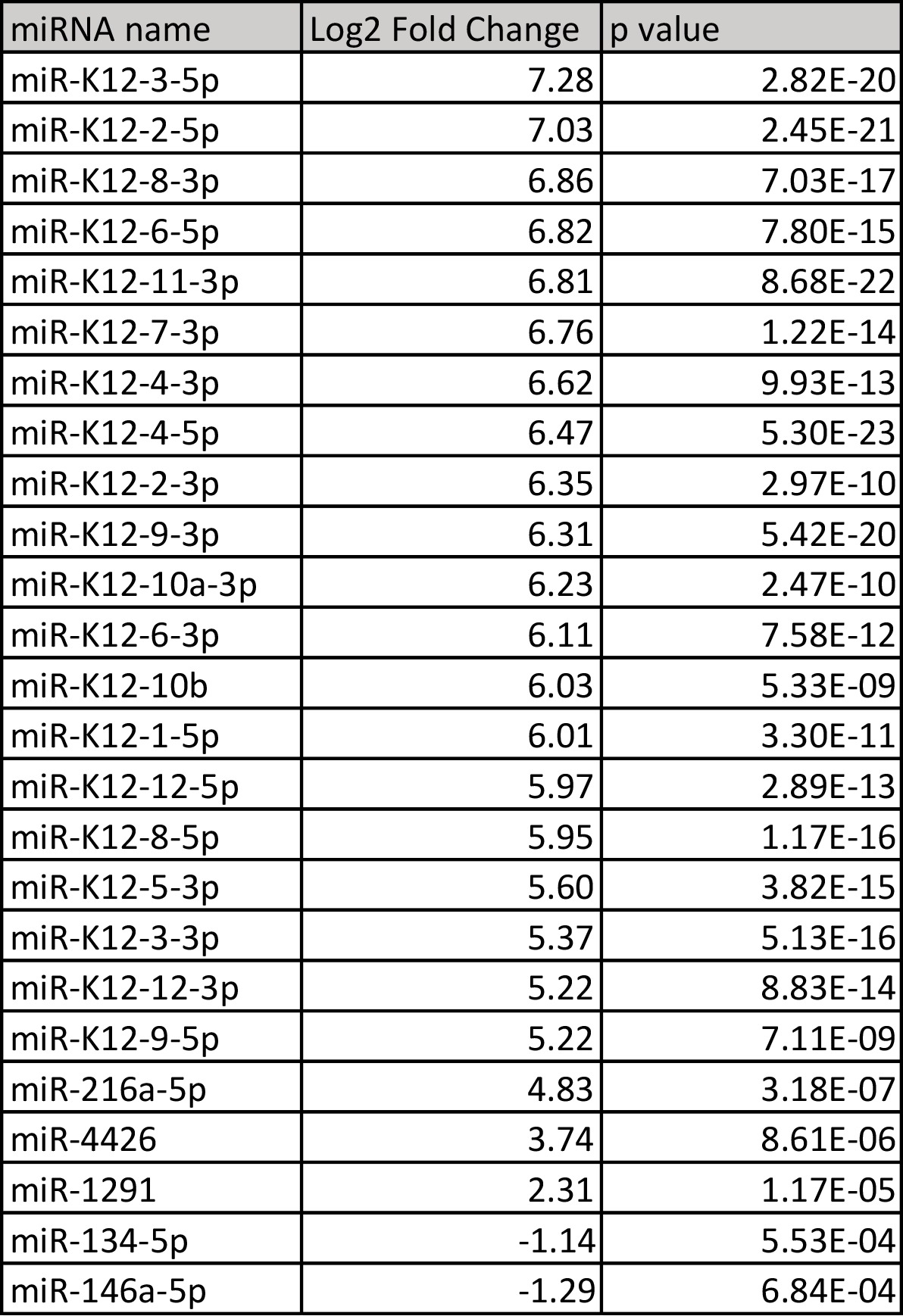

Supplement: S3 Table — (TIF) [file ppat.1006524.s009.tif]
